# Investigation into the Nucleation of the *p*-Hydroxybenzoic Acid:Glutaric Acid 1:1 Cocrystal from Stoichiometric and Non-Stoichiometric Solutions

**DOI:** 10.1021/acs.cgd.2c01522

**Published:** 2023-09-01

**Authors:** Hannah McTague, Åke C. Rasmuson

**Affiliations:** † Synthesis and Solid State Pharmaceutical Centre (SSPC), Bernal Institute, Department of Chemical and Environmental Science, University of Limerick, Limerick V94 T9PX, Ireland; ‡ Department of Chemical Engineering and Technology, KTH Royal Institute of Technology, Stockholm SE-100 44, Sweden

## Abstract

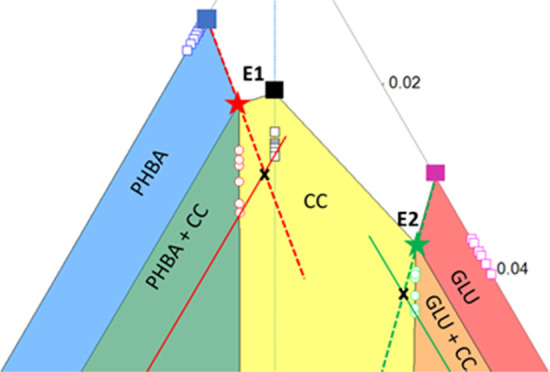

The nucleation in the *p*-hydroxybenzoic acid:glutaric acid 1:1 cocrystal (PHBA:GLU) system has been investigated in stoichiometric and non-stoichiometric acetonitrile solutions by induction time experiments. Utilizing the ternary phase diagram, the supersaturated non-stoichiometric solutions were created with compositions along the invariant point boundary lines. In all cases, the PHBA:GLU cocrystal was the nucleating phase, even though the non-stoichiometric solutions were also supersaturated with respect to the pure solid phases. The nucleation of the cocrystal from the mixed solutions is found to be more difficult than the nucleation of the pure compounds from the respective pure solutions, as captured by lower pre-exponential factors (*A*). However, if the driving force is defined per reactant molecule instead of per heterodimer, the cocrystal nucleation difficulty is close to that of the more difficult-to-nucleate pure compound. The difference in nucleation difficulty of the cocrystal from stoichiometric and non-stoichiometric solutions was captured by differences in the interfacial energy, while the pre-exponential factor remained unchanged. Apart from the pure GLU system, the relation between the experimentally determined pre-exponential factors for the different systems correlates with calculated values using theoretical expressions for volume-diffusion and surface-integration control.

## Abstract 2

The nucleation in the p-hydroxybenzoic acid:glutaric acid 1:1 cocrystal (PHBA:GLU) system has been investigated in stoichiometric and nonstoichiometric acetonitrile solutions by induction time experiments.

## Introduction

Crystallization is a key element of most industrial chemical processes, particularly in the pharmaceutical sector, as it represents a means of purification and separation of chemical products.^1^ For clinical and legal reasons, it is vital that high importance is given to the crystal form chosen,^2^ and therefore, there is a want for a greater variety of the number of crystalline forms available for an active pharmaceutical compound (API).^3^ The nature of APIs in the fact that they consist of molecules or ions with exterior functional groups that may engage in hydrogen bonding means that all APIs are candidates for cocrystals, offering an advantage over traditional types such as polymorphs and solvates which rely on high-throughput screening rather than design and over salts, which have the extra requirement of an ionizable functional group.^4^ Cocrystals have received much attention in recent years due to their potential to offer improved physicochemical properties of an API^5−7^ and different methods of cocrystal synthesis have been reported in the literature.^8−11^

In order to manufacture cocrystals at an industrial scale, knowledge of crystallization kinetics is an invaluable tool for process optimization, purity, and particle size distribution control. The nucleation kinetics of APIs can be studied by means of induction time experiments,^12^ providing information on the nucleation rate and determination of nucleation parameters such as interfacial energy and pre-exponential factors. To date, there has been little reported on the rate of nucleation of cocrystals and the dependence on solution composition. Furthermore, there are few studies in which cocrystal nucleation is rationalized against the nucleation of the pure compounds.^13^

For the design of the process for manufacturing cocrystals, a phase diagram over the systems is very helpful in detailing the stability regions for the various crystalline phases in a specific solvent^14^ and has been determined for a number of different cocrystal systems.^15,16^ The choice of solvent has been shown to have a great effect on the ternary phase diagrams (TPDs) of cocrystals and is explored in the literature.^41,42^ The phase diagrams of a 1:1 trans-cinnamic acid:nicotinamide cocrystal in different solvents were analyzed to explain why crystallization from a solution of stoichiometric amounts of each component sometimes formed pure cocrystal and sometimes did not depending on the solvent.^16^ TPDs can also be utilized to offer further insights into the kinetics of cocrystal formation and nucleation. A study of caffeine:maleic acid cocrystals using previously determined TPDs explored the effect of the solvent in the crystallization of stoichiometrically different cocrystals. This work also revealed that choosing the optimum solvent can enable the isolation of previously kinetically inaccessible metastable phases.^17^ The nucleation of a caffeine:glutaric acid system in acetonitrile has also been investigated through the use of a phase diagram to identify operating regions for cooling crystallization in order to optimize cocrystal purity.^18^ Croker et al. investigated the solid-phase nucleation in different regions of the TPD of the *p*-toluenesulfonamide:triphenylphosphine oxide cocrystal, which is stable as 1:1 and 3:2 cocrystals. It was concluded that kinetic factors influence the form of the nucleating phase, which can lead to the initial appearance of a phase different from that thermodynamically predicted by the TPD.^19^

The nucleation of *p*-hydroxybenzoic acid, a polymorphic API, has been investigated, revealing that a decrease in the required nucleation driving force correlated with decreasing solvent viscosity and increasing solubility, factors which go hand-in-hand with the pre-exponential component of the classical nucleation theory (CNT) expression.^20^ The utility of TPDs in the study of polymorphic cocrystal systems has been described in the literature, creating a platform to guide the production of polymorphic forms.^21−24^ The nucleation processes of a polymorphic API, spironolactone, were examined utilizing ternary phase diagrams in conjunction with induction time experiments to examine how the solid form is controlled not only by thermodynamics but also by kinetics.^25^

In the present work, the nucleation of *p*-hydroxybenzoic acid:glutaric acid 1:1 cocrystal (PHBA:GLU) in stoichiometric and non-stoichiometric solutions has been investigated. The composition of non-stochiometric solutions corresponds to solutions along the cocrystal invariant point boundary lines. The nucleation of the PHBA:GLU cocrystal is compared with the nucleation of the pure components.

**Figure 1:**
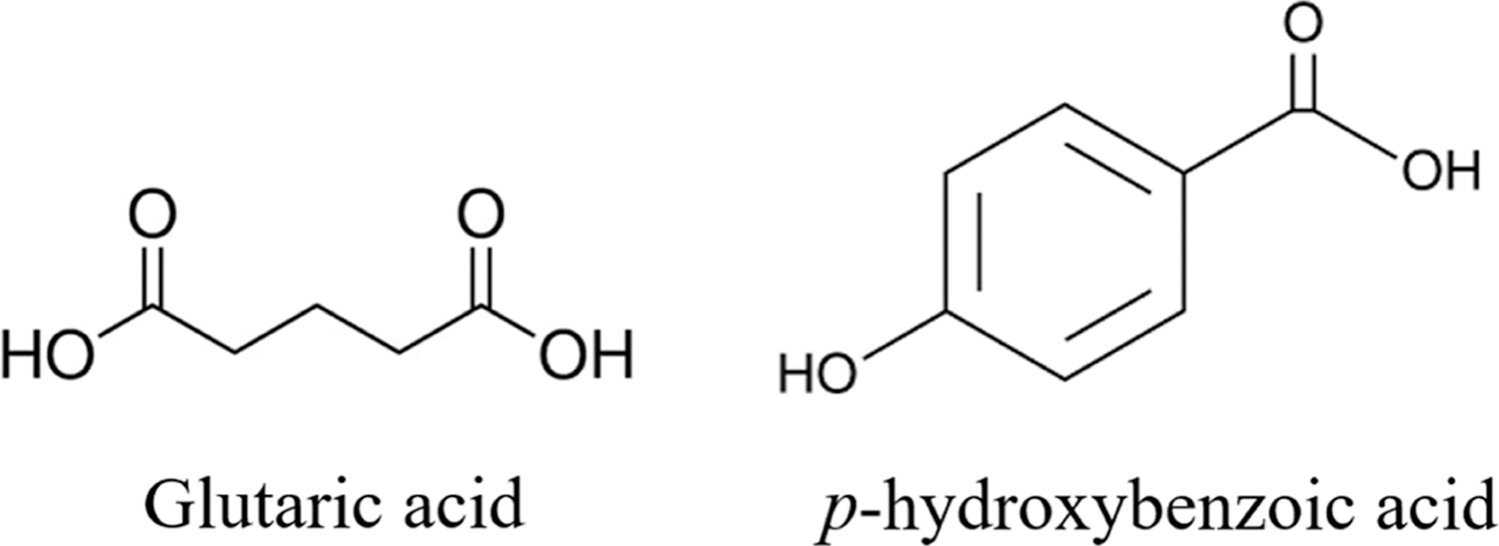
Glutaric acid and *p*-hydroxybenzoic acid.

*p*-Hydroxybenzoic acid (Figure 1) is a monohydroxybenzoic acid primarily used for the preparation of parabens, widely used as cosmetic and pharmaceutical preservatives due to its bactericidal and anti-fungal properties. The supramolecular synthon capability of PHBA makes it an important molecule to improve the physicochemical properties of APIs to enhance bioavailability. There are two polymorphs of *p*-hydroxybenzoic acid reported currently.^26−28^ The thermodynamics of *p*-hydroxybenzoic acid in a variety of solvents has been investigated previously.^29^ Glutaric acid (Figure 1) is a pentanedioic acid with two known polymorphs: the stable β polymorph and a metastable α polymorph and is commonly used as a cocrystal coformer.^30,31^ Glutaric acid has been shown to significantly increase the bioavailability and stability of some APIs when used as a cocrystal coformer, and it is a GRAS (generally recognized as safe) substance.^31^ The unit cell of the PHBA:GLU 1:1 cocrystal is given in the Supporting Information (SI). The TPDs for PHBA:GLU in acetonitrile (MeCN) have been determined^32^ and are used in the present study. The nucleation of β-glutaric acid has been investigated previously by means of induction time experiments in chloroform at 10 °C. From a stoichiometric solution of theophylline and glutaric acid, β-glutaric acid nucleates as a metastable form and subsequently transforms to the stable theophylline:glutaric acid 1:1 cocrystal. The nucleation of β-glutaric acid displayed shorter induction times from a mixed solution containing theophylline versus the pure solution when supplied with the same supersaturation driving force.^33^

## Experimental Section

**Figure 2:**
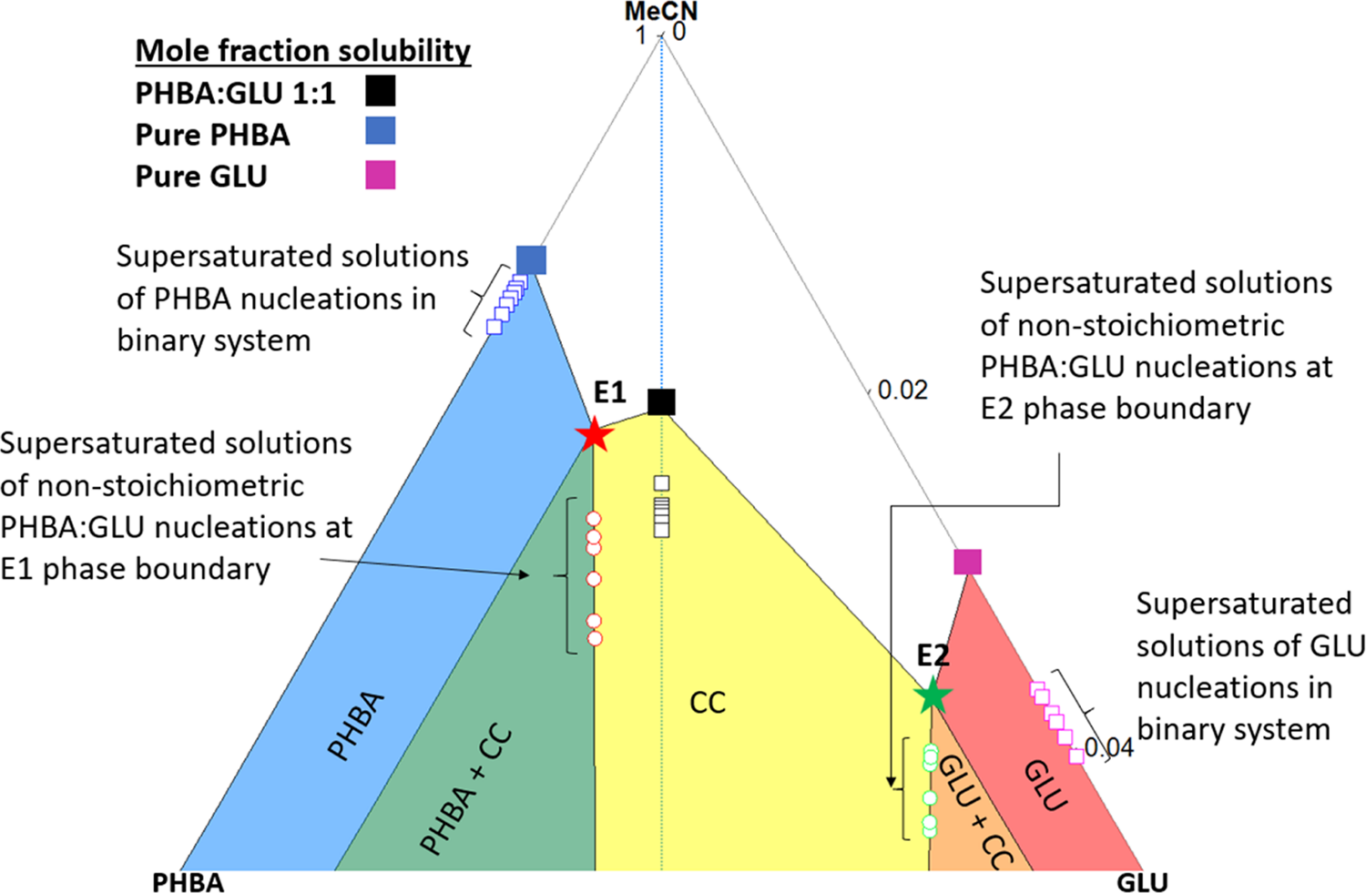
TPD in mole fractions for the PHBA:GLU 1:1 cocrystal in MeCN at 20 °C. White circles on the stoichiometric line represent the solution compositions of the 1:1 nucleation experiments. The invariant points E1 and E2 are marked by red and green stars, respectively. “CC” is the PHBA:GLU 1:1 cocrystal.

The experimental work includes solubility determination of the PHBA:GLU 1:1 cocrystal in equilibrium with stoichiometric and non-stoichiometric solutions, along with physical characterization of solid phases. Induction time experiments were performed on the cocrystal system from stoichiometric and non-stoichiometric solutions as well as on the individual cocrystal components from pure solutions. Non-stoichiometric solutions were created with a composition along the so-called invariant point boundary lines, i.e., the phase boundary lines of the region where the cocrystal is stable, as illustrated in Figure 2.

### Materials

Glutaric acid (GLU, CAS Registry Number 110-94-1) of purity ≥99% was used as received from Sigma-Aldrich. *p*-Hydroxybenzoic acid was used as received from Merck (PHBA, CAS Registry Number 00-96-7) with purity ≥99%. Acetonitrile was received from Fisher and was HPLC grade (CAS Registry Number 75-05-8).

### Preparation of the PHBA-GLU Cocrystal

Equimolar amounts of PHBA and GLU were added to acetonitrile and slurried for 48 h at 20 °C at 200 rpm. The solid was then filtered and characterized by powder X-ray diffraction (PXRD) and differential scanning calorimetry (DSC).

### Solubility

The solubility in grams of solute per gram of the solvent of the PHBA:GLU 1:1 cocrystal in equilibrium with a stoichiometric solution in acetonitrile at 10, 20, and 40 °C and non-stoichiometric solutions in acetonitrile at 20 °C was obtained by the well-reported gravimetric method and the details are presented in the SI. For non-stochiometric systems, the total dissolved mass in the supernatant in equilibrium with the solid phase after the nucleation experiments was determined gravimetrically and was combined with the invariant point relative composition reported in the literature.^32^ Solution samples were extracted from solutions that underwent nucleation in the nucleation experiments. The vials were left at the nucleation temperature for 1 week after nucleation to ensure equilibrium had been reached, and the gravimetric method was used to find the concentration. A sample of the solid phase in equilibrium was also taken and analyzed by PXRD.

The details of the solid-phase characterization techniques employed in this work including PXRD, DSC, and scanning electron microscopy (SEM) are presented in the SI.

### Nucleation Experiments

Induction time experiments have been performed at a nucleation temperature (*T*_nuc_) of 20 °C at a 20 mL scale as per previous work.^33^ A range of supersaturations (*S*) were created. Supersaturation, *S*, was calculated as a ratio of mole fraction concentration in the supersaturated solution, *X*, versus mole fraction concentration at equilibrium at *T*_nuc_, X*.
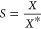
1In the case of the cocrystal, supersaturation (*S*) is defined as per our previous study
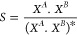
2*X*^*A*^ is the mole fraction concentration of PHBA and *X*^*B*^ is the mole fraction concentration of GLU. The denominator is the product of the mole fraction concentrations of PHBA and GLU at equilibrium. One mole of the PHBA:GLU 1:1 cocrystal is defined as an assembly consisting of one mole of PHBA and one mole of GLU.

To create the starting solutions for each set of induction time experiments for PHBA:GLU, PHBA, and GLU systems, *X* g of the solid was added to 320 g of acetonitrile to create desired supersaturation at *T*_nuc_ = 20 °C. The solid was dissolved at 50 °C, which was above the saturation temperature to ensure total dissolution. Following agitation at 400 rpm for 24 h, this solution was carefully and quickly filtered, with a heated apparatus, as 20 mL samples into 20× 30 mL glass vials. Vials were sealed tightly with a poly(tetrafluoroethylene) (PTFE) coated screw lid. The 20 mL samples were then left at the same dissolution temperature for another 24 h period. After this, the samples are submerged at 20 °C to create supersaturation and immediate recording with a HD video camera begins. The induction time is the amount of time between submersion at nucleation temperature, 20 °C (*T*_nuc_), and the first signs of nucleation visible to the naked eye of the video recordings. The procedure was repeated for each batch of vials, by which approximately 40 experiments were performed per condition for each system; see Table 1.

**Table 1:** Compositions in the Mole Fraction of the Solutions Used in Induction Time Experiments^a^

PHBA:GLU 1:1					
composition		composition at *T*_nuc_		(*X*^AB^)^2^/(*X*^AB^*)^2^	
PHBA	GLU	PHBA	GLU	*S*	*RT* ln *S*
0.0125	0.0125	0.0104	0.0104	1.45	906
0.0131	0.0131	0.0104	0.0104	1.59	1130
0.0132	0.0132	0.0104	0.0104	1.61	1161
0.0133	0.0133	0.0104	0.0104	1.64	1206
0.0134	0.0134	0.0104	0.0104	1.66	1235
0.0136	0.0136	0.0104	0.0104	1.72	1322

non-stoichiometric solutions on the E1 phase boundary line					
composition		composition at *T*_nuc_		(*X*^A^·*X*^B^)/(*X*^A^·*X*^B^)*	
PHBA (*X*^A^)	GLU (*X*^B^)	PHBA	GLU	*S*	*RT* ln *S*
0.0168	0.0102	0.0147	0.0077	1.51	1004
0.0173	0.0107	0.0147	0.0077	1.64	1206
0.0176	0.0110	0.0147	0.0077	1.72	1322
0.0185	0.0119	0.0147	0.0077	1.94	1615
0.0196	0.0131	0.0147	0.0077	2.27	1998

non-stoichiometric solutions on the E2 phase boundary line					
composition		composition at *T*_nuc_		(*X*^A^·*X*^B^)/(*X*^A^·*X*^B^)*	
PHBA (*X*^A^)	GLU (*X*^B^)	PHBA	GLU	*S*	*RT* ln* S*
0.0069	0.0330	0.0053	0.0316	1.36	749
0.0071	0.0332	0.0053	0.0316	1.40	820
0.0073	0.0334	0.0053	0.0316	1.45	906
0.0083	0.0343	0.0053	0.0316	1.69	1279
0.0090	0.0350	0.0053	0.0316	1.86	1512
0.0092	0.0352	0.0053	0.0316	1.93	1603

a Equilibrium compositions at *T*_nuc_ are also shown. *S* is the supersaturation with respect to the PHBA:GLU cocrystal and *RT* ln *S* is the driving force in J mol^–1^.

For the non-stoichiometric nucleation studies, the supersaturation was calculated by eq 2 where the solubility product in the denominator is the product of the mole fraction concentrations of PHBA and GLU in a solution saturated by the cocrystal at the points E1 and E2, respectively.

## Results and Analysis

### Solid-Phase Characterization

The PHBA:GLU cocrystal synthesized was identified through PXRD as the previously reported form.^32^ In induction time experiments of the PHBA:GLU system in stoichiometric and non-stoichiometric solutions, the only solid phase nucleating was the 1:1 PHBA:GLU cocrystal as is supported by diffractograms presented in the SI. An endotherm was detected by DSC, representing the melting of the cocrystal at 133 °C, which is in accordance with previously reported data.^32^ The DSC also shows a slight second thermal event at 136 °C; see the SI. However, this was not investigated further. The melting points of the pure components GLU and PHBA are 97 and 215 °C, respectively; DSC profiles can be found in the SI. SEM micrographs reveal that the cocrystal forms rectangular block-shaped crystals when crystallized in MeCN at 20 °C. In induction time experiments performed on the pure components, the nucleating solid in the PHBA system was identified as CSD ref code JOZZIH01^27^ and in the GLU system as β-glutaric acid (CSD ref code GLURAC04).^34^ Further details, powder diffractograms, scanning calorimetry profiles, and micrographs are given in the SI file.

### Solubility

The cocrystal phase boundary lines plotted on the TPD in Figure 2 are straight lines drawn from the invariant point E1 or E2 to the 1:1 point on the binary solid system axis of PHBA-GLU and are referred to as the E1 phase boundary line and the E2 phase boundary line, respectively. These “boundary” lines are tie lines coinciding with the boundary of the cocrystal region. Points were selected along these lines providing ternary compositions in mole fractions (Table 2) for PHBA, GLU, and MeCN used to create supersaturated solutions for non-stoichiometric nucleation experiments.

**Table 2:** Solubility of the PHBA:GLU Cocrystal in Equilibrium with Stoichiometric and Non-Stoichiometric Solutions Determined by the Gravimetric Method

PHBA:GLU cocrystal solubility			
solution	g g^–1^ MeCN	no. experiments	std. dev.
1:1 (10 °C)	0.0459	3	0.0005
1:1 (20 °C)	0.0700	4	0.0004
1:1 (40 °C)	0.1735	3	0.0004
E1 (20 °C)	0.0751	5	0.0001
E2 (20 °C)	0.1238	8	0.0005

In Figure 2, the colored regions on the TPD represent the mole fraction compositions where different solid phases are stable in equilibrium with the solution. The region CC is where the pure 1:1 PHBA:GLU cocrystal is the stable solid phase; the liquid–solid-phase boundary of this region lies between the invariant points, E1 and E2. The width of the cocrystal region is calculated from the 3D distance between points E1 and E2. The cocrystal region widths from the PHBA:GLU TPDs at 2, 20, and 40 °C provided by Yang et al.^32^ have been calculated and are presented in the SI. The width of the cocrystal region decreases as the equilibrium temperature is decreased from 40 to 20 to 2 °C. Although the TPD at 40 °C has the widest cocrystal region, which may be attractive from a processing point of view, this investigation is performed utilizing the 20 °C TPD since the solubility of β-GLU is very high at 40 °C (Table 2), and vast quantities of materials would be needed for the induction time experiments.

The solubility of the PHBA:GLU cocrystal in equilibrium with a stoichiometric solution 1:1 at 10, 20, and 40 °C and in equilibrium with non-stoichiometric solutions, E1 and E2, at 20 °C in MeCN is presented in Table 2.

**Figure 3:**
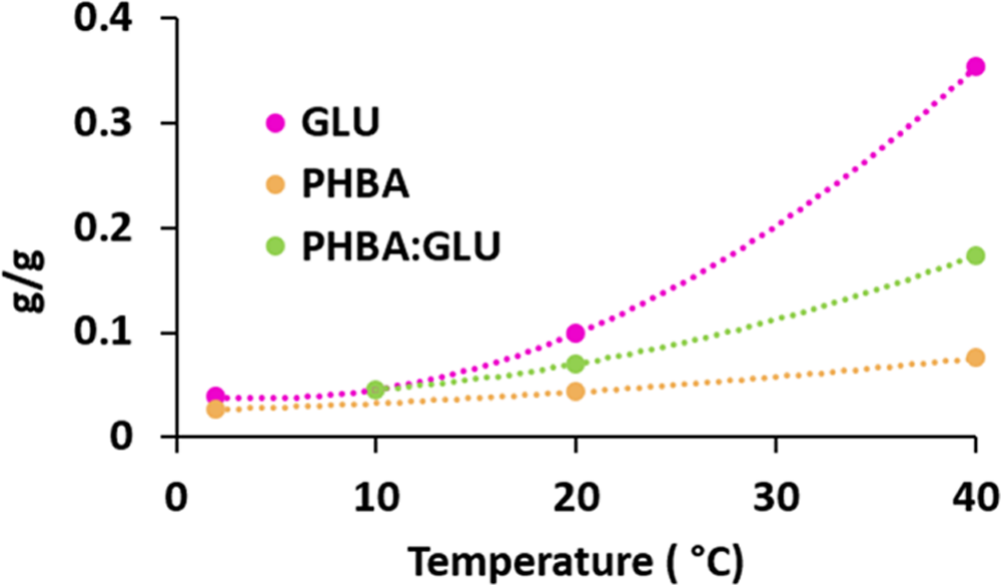
Solubility (g g^–1^ solvent) in MeCN of the PHBA:GLU cocrystal in the stoichiometric solution and of pure PHBA and β-GLU.

The solubilities of the pure components PHBA and GLU have been determined previously^32^ and are included in Figure 3 for comparison with the PHBA:GLU cocrystal in a stoichiometric solution. The g g^–1^ solubility of the cocrystal is higher than that of pure PHBA and lower than that of β-GLU.

The corresponding g g^–1^ concentration of PHBA and GLU in the stoichiometric equilibrium solution is presented in Table 3, along with the solubility of PHBA:GLU and pure PHBA and GLU in mole fractions. In the nucleation experiments of the cocrystal, from solutions along the invariant point boundary lines, the liquid-phase equilibrium composition corresponds to E1 and E2. The mole fraction solution compositions at E1 and E2 have been determined previously^32^ and are also shown in Table 3. There is excellent agreement between the g g^–1^ values determined by the gravimetric method for E1 and E2 (second column Table 3) and the literature values^32^ for the solubility of the cocrystal at E1 and E2, which equate to 0.0760 and 0.1239 g g^–1^, respectively.

**Table 3:** Solubility in MeCN at 20 °C of the PHBA:GLU Cocrystal in Stoichiometric (1:1) Solution and Non-Stoichiometric Solution Compositions of the Invariant Points E1 and E2 and of Pure PHBA and GLU

	total solid mass from gravimetric method	mole fraction		g g^–1^ MeCN	
	g g^–1^ MeCN	PHBA	GLU	PHBA	GLU
1:1	0.0700	0.0104	0.0104	0.0358	0.0342
E1	0.0751	0.0147^a^	0.0077^a^	0.0501	0.0250
E2	0.1238	0.0053^a^	0.0315^a^	0.0186	0.1052
PHBA		0.0127^a^		0.0433	
GLU			0.0298^a^		0.0989
solubility product (MF)	**1:1**	**E1**		**E2**	
	0.000108	0.000113		0.000166	

a Data from Yang et al.^32^

The solubility product of the cocrystal (Table 3) is increased in the non-stoichiometric systems versus the stoichiometric system and is the greatest in the E2 non-stoichiometric solution. Compared to the corresponding pure binary systems, the solubility of solid PHBA is higher in the E1 solution and the solubility of solid β-GLU is higher in the E2 solution. The solubilities in mol L^–1^ of the cocrystal and pure components at 20 °C are shown in the SI.

### Nucleation

The nucleating solid was identified as the PHBA:GLU cocrystal in all samples filtered from the nucleation experiments at stoichiometric and non-stoichiometric conditions and PXRDs can be found in the SI. For stoichiometric solutions, this includes samples from 30 s to 8 h post-nucleation. The non-stoichiometric experiments have been performed with compositions along the E1 and E2 phase boundary lines, as shown on the TPD in Figure 2 referred to as “non-stoichiometric solutions E1 and E2.”

Induction time (τ) probability distributions have been fitted by the Poisson distribution eq 3 ^35^
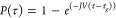
3where *V* is the sample volume, τ is the induction time, τ_g_ is the nucleus growth time, and τ is the individual induction time measurement. The Poisson distribution fits reasonably well to all data with coefficients of determination (*R*^2^) values >0.9 in most cases. All induction time probability distributions, *P*(τ), for the PHBA:GLU cocrystal from stoichiometric and non-stoichiometric solutions, as well as for PHBA and β-GLU from the respectively pure solutions, all at 20 °C, along with fittings of the Poisson distribution (eq 3) are presented in the SI.

The growth times, i.e., the τ_g_ values, extracted by fitting eq 3, were negative in most cases for the cocrystal in the stoichiometric solution and in some cases for the cocrystal in non-stoichiometric and for PHBA, as shown in the SI. Accordingly, the time of the first induction time point is hereon instead taken as the growth time, τ_g_. As shown in the SI, these τ_g_ values are quite scattered, and for smoothing purposes, an exponential function was fitted, from which the τ_g_ values used are extracted for each condition. The nucleation time, τ_nuc_, is calculated as the median induction time, τ_50_, minus the growth time, τ_g_. The data extracted from the induction time experiments of all systems are presented in Table 4.

**Table 4:** Median Induction Times, τ_50_ (s), Obtained from Experimentally Determined P(τ)s over a Range of Supersaturation Ratios, *S*, with Corresponding Driving Forces (*RT* ln *S*) for PHBA:GLU, PHBA, and GLU Systems^a^

PHBA:GLU								PHBA								β-GLU							
*S*	*RT* ln *S* (J mol^–1^)	τ_50_ (s)	CV	τ_g_ (s)	τ_g_ (%)^d^	τ_nuc_ (s)	*J*^e^ (m^–3^ s^–1^)	*S*	*RT* ln *S* (J mol^–1^)	τ_50_ (s)	CV	τ_g_ (s)	τ_g_ (%)^d^	τ_nuc_ (s)	*J*^e^ (m^–3^ s^–1^)	*S*	*RT* ln *S* (J mol^–1^)	τ_50_ (s)	CV	τ_g_ (s)	τ_g_ (%)^d^	τ_nuc_ (s)	*J*^e^ (m^–3^ s^–1^)
**1.45**	906	3473	1.09	311	9	3162	16	**1.08**	188	8593	0.96	690	8	7903	6	**1.22**	485	3668	0.62	817	22	2851	18
**1.59**	1130	2435	1.32	245	10	2190	23	**1.11**	254	2405	1.52	534	22	1871	27	**1.24**	524	2347	0.80	295	13	2052	24
**1.61**	1161	2000^b^	1.00	237	12	1763	28	**1.13**	298	1765	0.77	450	25	1315	38	**1.27**	583	2265	0.93	323	14	1942	26
**1.64**	1206	1628	1.26	226	14	1402	36	**1.15**	341	1059	0.93	379	36	680	74	**1.29**	621	1624	1.63	545	34	1079	46
**1.66**	1235	1470	1.31	218	15	1252	40	**1.18**	403	980	2.09	293	30	687	73	**1.31**	658	1121^c^	1.15	209	19	912	55
**1.72**	1322	1088	1.03	197	18	891	56	**1.22**	485	562	0.81	209	37	353	141	**1.35**	731	1074	0.62	294	27	780	64

E1								E2							
*S*	*RT* ln *S* (J mol^–1^)	τ_50_ (s)	CV	τ_g_ (s)	τ_g_ (%)^d^	τ_nuc_ (s)	*J*^e^ (m^–3^ s^–1^)	*S*	*RT* ln *S* (J mol^–1^)	τ_50_ (s)	CV	τ_g_ (s)	τ_g_ (%)^d^	τ_nuc_ (s)	*J*^e^ (m^–3^ s^–1^)
**1.51**	188	4040	1.19	227	6	3813	13	**1.36**	485	4708	1.06	403	9	4305	12
**1.64**	254	2718	0.68	217	8	2501	20	**1.40**	524	3964	0.90	540	14	3424	15
**1.72**	298	2015	1.41	210	10	1805	28	**1.45**	583	3094	1.08	606	19	2513	20
**1.94**	341	905	1.44	193	21	712	70	**1.69**	621	1660	0.97	285	17	1375	36
**2.27**	403	828	1.44	171	21	657	76	**1.86**	658	860	1.17	150	17	710	70
								**1.93**	731	630	1.22	166	26	464	108

a Coefficient of variation (CV) (standard deviation/mean) calculated to describe the spread of nucleation induction times.

b τ_50_ estimated directly from experimental data.

c τ_50_ as per Poisson fit to data.

d τ_g_ % is the percentage of the growth time relative to τ_50_.

e Nucleation rates, *J*, estimated from the nucleation time according to eq 4.

As is expected, τ_50_ decreases with increasing supersaturation for all systems in Table 4, and so does τ_nuc_. The percentage of τ_g_ relative to the median induction times is the lowest for the PHBA:GLU cocrystal.

**Figure 4:**
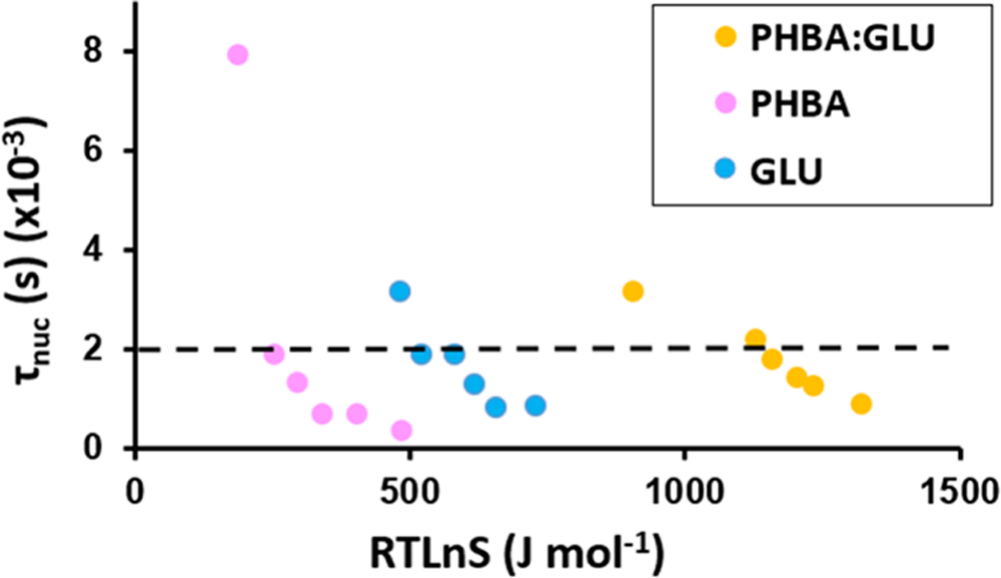
Nucleation times for PHBA:GLU, pure PHBA, and pure β-GLU systems at different driving forces.

In Figure 4, the nucleation time τ_nuc_ is plotted versus driving force (*RT* ln *S*) for PHBA:GLU nucleation from a stoichiometric solution, PHBA from a pure solution, and β-GLU from a pure solution. The driving force required to achieve the same nucleation time is used to describe the “nucleation difficulty.”

**Figure 5:**
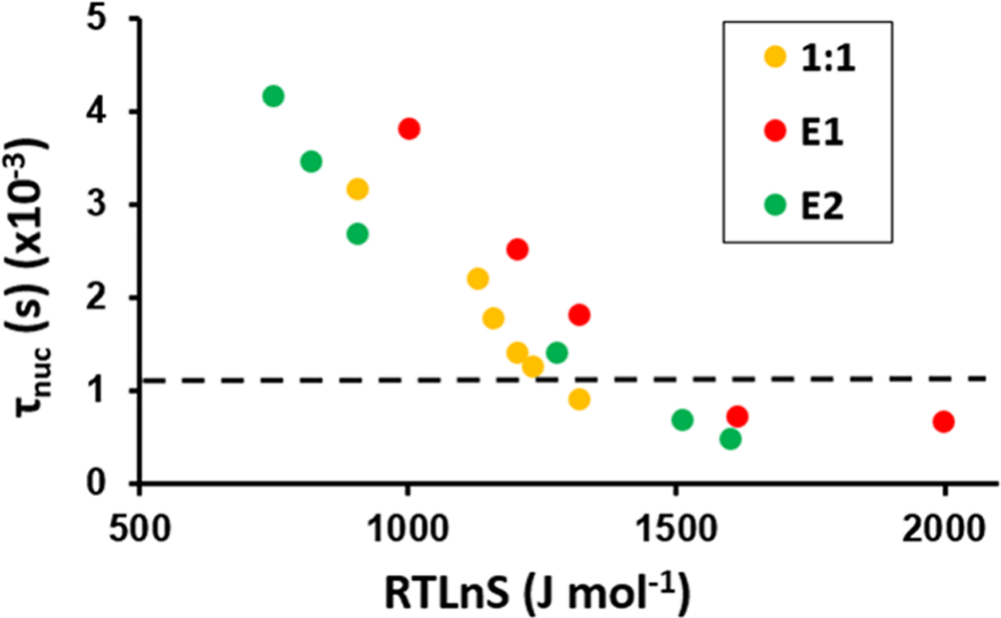
τ_nuc_ (nucleation time) versus driving force (RT ln *S*) for PHBA:GLU nucleation from a stoichiometric 1:1 solution and non-stoichiometric solutions with compositions on the E1 and E2 phase boundary lines.

In order to nucleate at the same time, a higher driving force is required for the PHBA:GLU cocrystal, followed by β-GLU from the pure solution, and PHBA from the pure solution nucleates with the greatest ease, i.e., the nucleation of the PHBA:GLU cocrystal from a stoichiometric solution is more “difficult” than the nucleation of the pure solids from respective pure solutions, which is similar to previous observations in the theophylline:salicylic acid 1:1 cocrystal (THP:SA) system.^13^ The nucleation times versus the driving force for PHBA:GLU nucleation from solutions of different compositions are compared in Figure 5.

As can be seen in Figure 5, the nucleation difficulty of the PHBA:GLU cocrystal from stoichiometric and non-stoichiometric E2 solutions is quite similar. Cocrystal nucleation from non-stoichiometric solutions along the E1 phase boundary line is slightly more difficult. For a τ_nuc_ of ∼2500 seconds, a driving force of 905 J mol^–1^ is required in the E2 system, 1050 J mol^–1^ in the 1:1 system, and 1205 J mol^–1^ in the E1 system.

The nucleation difficulty of the different systems follows the same order as the growth times, τ_g_. Even if the growth time data are scattered, Figure 10 shows that to reach the same growth time, the lowest driving force is required for PHBA, followed by GLU. The slowest growth is found in the cocrystal systems.

### Nucleation Kinetics

The nucleation time, τ_nuc_, is related to the nucleation rate, *J*, by eq 4
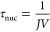
4where the volume is 20 mL. The nucleation rates calculated are presented in Table 4. Overall nucleation rates are in the range of 6–141 m^–3^ s^–1^.

As in previous work,^13^ the induction time results have been analyzed within the classical nucleation theory for determination of the interfacial energy and the pre-exponential factor using the relation
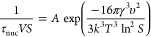
5where *V* is the liquid volume of 20 mL in the present work, *A* is a pre-exponential factor (m^–3^ s^–1^), *R* is the universal gas constant (J K^–1^ mol^–1^), *T* is the temperature (K), γ is the interfacial energy (J m^–2^), υ is the molecular volume (m^3^), and *k* is the Boltzmann constant (J K^–1^).

For a CNT plot, ln(τ_nuc_*S) is plotted against ln *S*^–2^*T*^–3^, and the interfacial energy is extracted from the slope of the line and the pre-exponential factor from the intercept. Obviously, the supersaturation, *S*, appears in both the abscissa and the ordinate; however, the origin differs, which is of importance in the case of a multicomponent system. The *S* value in the *y*-coordinate (left-hand side of eq 5) originates from a transformation of solute concentration, *C*, into S*C_e_. This “concentration” appears in the analysis of the pre-exponential factor^36^ and specifically of the attachment frequency. When the attachment frequency is controlled by volume diffusion, it is the product of the diffusion flux to the nucleus surface and the surface area, where the diffusion flux is simply the concentration times the diffusivity divided by the nucleus radius, as obtained by solving for diffusion in a stagnant solution of spherical geometry. For nucleation of a multicomponent solid of neutral compounds in a reasonably dilute solution, the flux that limits the formation of the nucleus is governed by the compound having the slowest transport rate, i.e., the lowest diffusivity times the corresponding concentration, D*C. Accordingly, the *S* value on the left-hand side of eq 5 is to be calculated for that. The transport limiting component is GLU in the E1 system, PHBA in the E2 system, and GLU in the 1:1 system.

**Figure 6:**
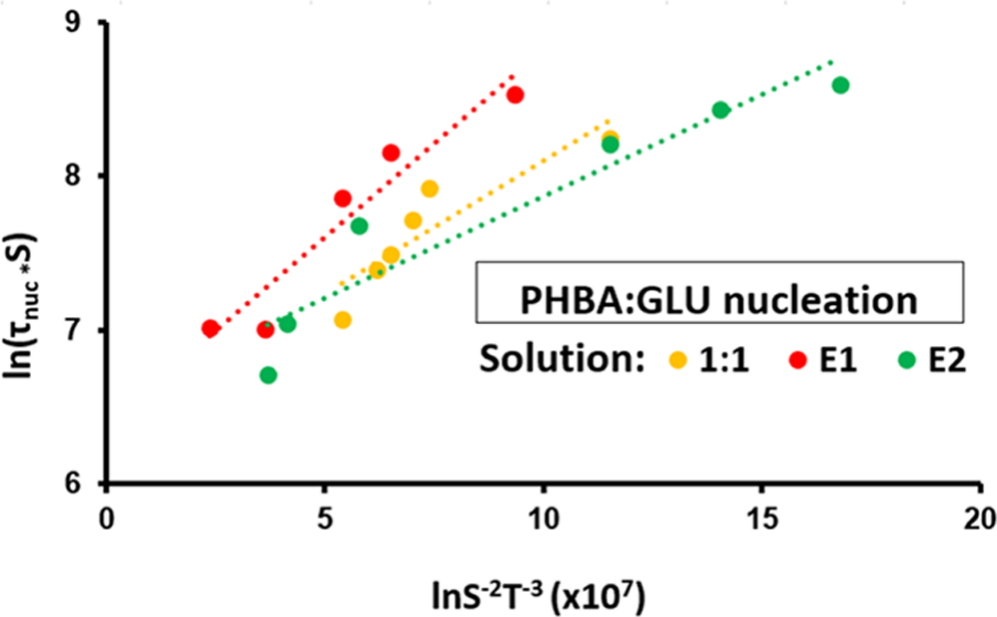
CNT plot for PHBA:GLU nucleation from a stoichiometric 1:1 solution and non-stoichiometric solutions with compositions on the E1 and E2 phase boundary lines.

In the exponential term in the *x*-coordinate, *RT* ln *S*, describes the free energy difference between molecules in the solution and in the crystalline solid phase. For a multicomponent solid, this free energy difference can be given per heterodimer in the 1:1 cocrystal or, e.g., per individual “reactant” molecule. Here, it is calculated per PHBA:GLU heterodimer assembly in accordance with eq 2. The molecular volume in the exponential term of eq 5 originates from the transformation of the free energy difference per unit volume of the nucleus to free energy difference per mole and is therefore related to the definition of *RT* ln *S*. Here, it is accordingly calculated as the molecular volume of the PHBA:GLU heterodimer. The CNT plot for PHBA:GLU nucleation from stoichiometric and non-stoichiometric solutions is presented in Figure 6.

**Figure 7:**
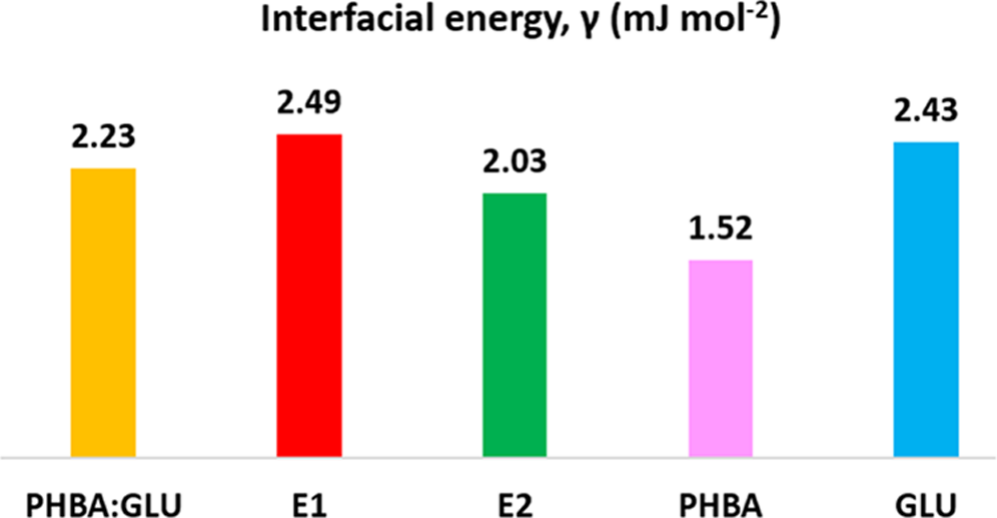
Interfacial energy, γ, in mJ mol^–2^ calculated from eq 5 for PHBA:GLU nucleation in a stoichiometric solution and non-stoichiometric solutions on the E1 and E2 phase boundaries. PHBA nucleation from a pure solution and β-GLU nucleation from a pure solution.

**Figure 8:**
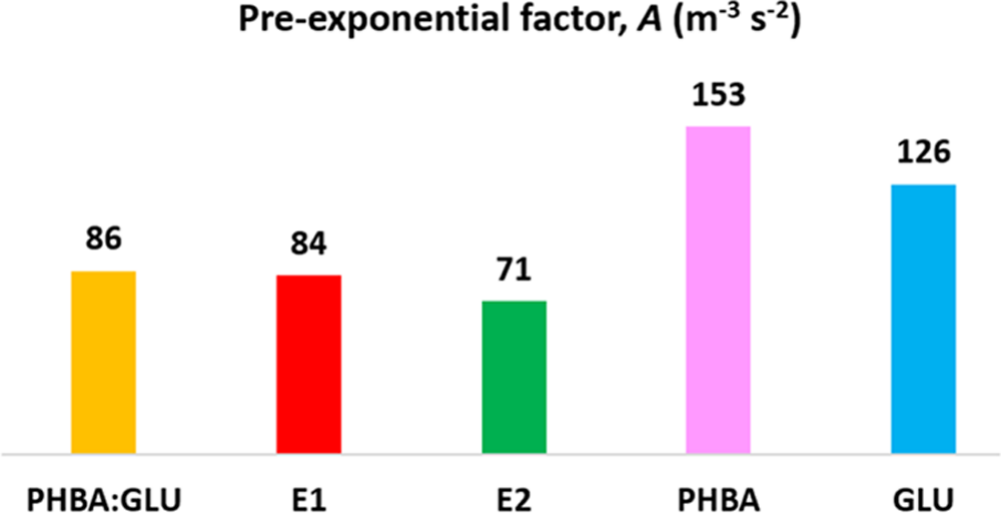
Pre-exponential factors, *A*, in m^–3^ s^–2^ calculated from eq 5 for PHBA:GLU nucleation in a stoichiometric solution and non-stoichiometric solutions on the E1 and E2 phase boundaries. PHBA nucleation from a pure solution and β-GLU nucleation from a pure solution.

The corresponding CNT plot for PHBA and β-GLU nucleation from the respective single solute solutions is presented in the SI. The interfacial energy and pre-exponential factor for all nucleation systems in this work are shown in Figures 7 and 8, respectively.

Obviously, the fact that the nucleation of the PHBA:GLU cocrystal appears to be more difficult compared to the nucleation of the pure compounds is primarily due to a lower pre-exponential factor, even though also the interfacial energy is high. The easy nucleation of PHBA can be attributed to it having the lowest interfacial energy and highest pre-exponential factor. The intermediate nucleation difficulty of β-GLU is due to a combination of high interfacial energy, which is the same as for the cocrystal, but an intermediate pre-exponential factor.

The difference in nucleation difficulty of PHBA:GLU from stoichiometric and non-stoichiometric solutions (Figure 5) is captured by differences in the interfacial energy (Figure 7). The nucleation difficulty of the cocrystal from the 1:1 solution and the non-stoichiometric E2 solutions were overall similar, while the nucleation from the E1 solution was somewhat more difficult. The interfacial energy is the highest in the latter solution and the lowest in the E2 solutions. Looking at how the solution composition changes going from E1 and 1:1 to E2 systems, the mole fraction ratio of PHBA:GLU in the E1 equilibrium solution is 1:0.52 and has the highest interfacial energy of 2.49 mJ m^–2^. The 1:1 system has an intermediate interfacial energy of 2.23 mJ m^–2^, and the E2 system has a mole fraction ratio of 1:5.94 in the equilibrium solution and the lowest interfacial energy of 2.03 mJ m^–2^. The data indicates that when there is more GLU available in solution, the creation of an interface for nucleation of the cocrystal becomes less thermodynamically unfavorable, and this is analyzed further in the Discussion section. The mole fraction solubility product of the cocrystal (Table 3) is very similar in E1 and 1:1 systems; however, it is increased in the E2 system, and this is in line with the lower interfacial energy.^36^ In the pure systems, the interfacial energy of PHBA nucleation has the lowest value of 1.52 mJ m^–2^, while the value for GLU is 2.43 mJ m^–2^, the latter being similar to the interfacial energy for the E1 cocrystal system.

### Analysis of Pre-Exponential Factors

Further analysis of the pre-exponential factor, *A*, is performed based on the previous work of Kashchiev and van Rosmalen.^36^ The pre-exponential factor is basically the product of the attachment frequency, the Zeldovich factor, and the concentration of nucleation sites. Since the concentration of nucleation sites is assumed equal to the inverse of the solute molecular volume, υ_o_, all molecular volumes in the derivation are the same and reduce into an inverse dependence in the final expression for A in the case of control by volume diffusion, and to an inverse dependence raised to 1/3 in the case of interphase-transfer control. However, for nucleation of a multicomponent solid phase, the analysis becomes more complex since the mass transfer limiting component may vary. In addition, there is a choice in how to define the free energy difference between the solution and the solid. As is discussed later, e.g., the free energy difference can be per heterodimer or per reactant molecule. Accordingly, three different molecular volumes are identified related to (i) how the concentration of nucleation sites is estimated, (ii) how the free energy difference between the solution and the solid is defined, and (ii) what component is limiting the mass transfer.

In the derivation by Kashchiev and van Rosmalen,^36^ the concentration of nucleation sites is set equal to the inverse of the molecular volume of the solute. However, in principle, every molecule in the solution can act as a nucleation site. In the present work, the concentration of nucleation sites is instead taken as the inverse of the solvent molecular volume, *V*_0_, since the solvent molecules are the most abundant. Thus, the concentration of nucleation sites becomes the same for all of the systems studied.

The molecular volume in the Zeldovich factor relates to how the free energy difference between the solution and the solid is defined as υ_0_. In the attachment frequency factor, the molecular volume, in the case of control by volume diffusion, originates from the nucleus size and thus has the same thermodynamic origin as in the Zeldovich factor, which leads to the conclusion that this parameter cancels out, leaving us with the expression given in eq 6. In the case of attachment controlled by interface transfer, the molecular volume, υ_MT_, in the expression for the flux in the attachment frequency factor originates from a description of the molecular jump of the mass transfer limiting molecule, while the nucleus surface area and the Zeldovich factor still carry the molecular volume related to the free energy difference. This leads to the conclusion that all three molecular volumes remain in the equation as given by eq 7.
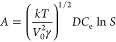
6
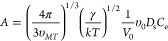
7The derivation of these two equations is detailed in the SI. For the calculations, *V*_o_ is the molecular volume of MeCN (0.86 × 10^–28^ m^3^), υ_MT_ depends on the composition of the solution, and υ_o_ depends on the definition of the free energy difference, all in units of m^3^. *D* is the diffusivity, *D*_s_ refers to the surface diffusivity^37,38^ (m^2^ s^–1^), γ is the interfacial energy (J m^–2^), *C*_e_ is the solubility (i.e., in the present work at 20 °C) in molar concentration (mol m^–3^), and *A* is obtained in m^–3^ s^–1^. *D* of the pure compounds had been estimated by the Wilke and Chang equation eq 8(37)
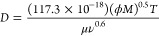
8where *D* is the diffusivity in m^2^ s^–1^, *M* is the molecular weight of the solvent in kg kmol^–1^, *T* is the temperature in K, μ is the solvent viscosity in kg m^–1^ s^–1^, *ν* is the solute molar volume in m^3^ kmol^–1^, and ϕ is the association factor for the solvent, which is 1 for acetonitrile. The diffusivity of GLU 1.015 × 10^–10^ m^2^ s^–1^ becomes very similar to that of PHBA 1.023 × 10^–10^ m^2^ s^–1^. As in the previous work, *D*_s_ is assumed equal to *D* mainly because of the lack in general of data for *D*_s_. The detailed derivations of eqs 7 and S7 are given in the SI.

The *C*_e_ parameter in eqs 6 and 7 originates from the representation of the mass transfer flux in the attachment frequency description, i.e., from the D*C term. For a multicomponent case of neutral molecules at low concentrations, this term represents the flux of the mass transfer limiting component. *C* = *C*_e_*S, and by including *S* in the *y*-coordinate of eq 5, the remaining *C*_e_ (mol m^–3^) is the concentration of the mass transfer liming component in a solution in equilibrium with the cocrystal solid phase. For PHBA:GLU cocrystal nucleation from the non-stoichiometric solutions, the components are being consumed stoichiometrically from the solution, and as crystallization progresses, the solution becomes depleted in PHBA and GLU and moves toward the invariant points, representing the equilibrium situation for the non-stoichiometric solutions studied in this work. The equilibrium concentration is therefore represented by the solution composition at E1 or E2 saturated with respect to the solid cocrystal. In nucleation of the cocrystal from non-stoichiometric solutions along the E1 phase boundary, the solution is rich in PHBA and lean in GLU; therefore, since the diffusivity of PHBA and GLU are quite similar, GLU is the mass transfer limiting molecule. For the same reasons, PHBA is the mass transfer limiting molecule in the E2 system.

As seen in Table 5, the *A* values calculated from eqs 6 and 7 are far higher than the experimental values in Figure 7, which is a common observation for this type of analysis.^13,33^ Experimentally, the pure systems have higher pre-exponential values than the cocrystal systems, and the values obtained from eqs 6 and 7 reflect this, although, for volume-diffusion control, the *A* values of PHBA are similar to the cocrystal systems. Notably, *A* obtained experimentally for PHBA is higher than that for β-GLU, which is opposite to the calculations according to eqs 6 and 7.

**Table 5:** Pre-Exponential Factors in Stoichiometric and Non-Stoichiometric Solutions: According to Equation 6, Volume-Diffusion-Controlled Nucleation, and According to Equation 7, Interface-Transfer Controlled Nucleation^a^

PHBA:GLU stoichiometric 1:1 (limiting: GLU)				β-GLU (binary system)			
*S*	Ce	A, volume-diffusion	A, interface-transfer	*S*	Ce	A, volume-diffusion	A, interface-transfer
**1.20**	202	5.98	8.33	**1.22**	586	17.75	24.89
**1.26**	202	7.47	8.33	**1.24**	586	19.20	24.89
**1.27**	202	7.67	8.33	**1.27**	586	21.33	24.89
**1.28**	202	7.96	8.33	**1.29**	586	22.73	24.89
**1.29**	202	8.16	8.33	**1.31**	586	24.10	24.89
**1.31**	202	8.73	8.33	**1.35**	586	26.78	24.89

PHBA:GLU non-stoichiometric E1 (limiting: GLU)				PHBA (binary system)			
*S*	Ce	A, volume-diffusion	A, interface-transfer	*S*	Ce	A, volume-diffusion	A, interface-transfer
**1.23**	150	4.65	6.56	**1.08**	245	3.67	10.15
**1.28**	150	5.58	6.56	**1.11**	245	4.97	10.15
**1.31**	150	6.12	6.56	**1.13**	245	5.82	10.15
**1.39**	150	7.48	6.56	**1.15**	245	6.66	10.15
**1.51**	150	9.25	6.56	**1.18**	245	7.88	10.15
				**1.22**	245	9.47	10.15

PHBA:GLU non-stoichiometric E2 (limiting: PHBA)			
*S*	Ce	A, volume-diffusion	A, interface-transfer
**1.17**	105	2.71	4.22
**1.18**	105	2.97	4.22
**1.20**	105	3.28	4.22
**1.30**	105	4.63	4.22
**1.36**	105	5.47	4.22
**1.39**	105	5.80	4.22

a *A* values for pure component nucleation in binary systems are also shown. *C*_e_ is in mol m^–3^ of the rate-limiting component and the *A* values shown are in units of ×10^10^ m^–3^ s^–1^.

## Discussion

### Nucleating Solid Phase

**Figure 9:**
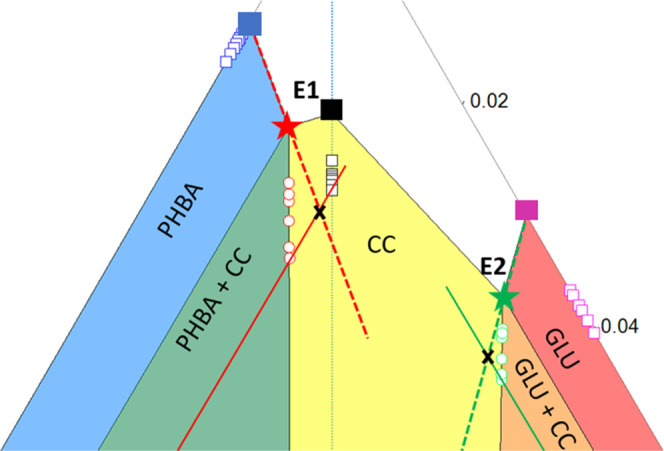
Ternary phase diagram (TPD) for the PHBA:GLU cocrystal system in MeCN at 20 °C. CC refers to the PHBA:GLU 1:1 cocrystal.

In the nucleation experiments from mixed solutions, it is always the 1:1 cocrystal that nucleates. In the experiments in stoichiometric solutions, the cocrystal is the only stable phase, while in the non-stoichiometric solutions with compositions along the E1 and E2 phase boundary lines, both the cocrystal and one of the pure components are stable solid phases. In the TPD in Figure 9, the different colored surfaces represent the different stability regions for the solid phases. The invariant points are marked E1 (red star) and E2 (green star). The compositions of the supersaturated solutions for the PHBA:GLU 1:1 nucleation experiments are shown as black outlined squares on the stoichiometric line, and the compositions of pure PHBA and pure β-GLU nucleation studies are shown as blue and pink outlined squares on the binary PHBA-MeCN and GLU-MeCN axes, respectively. As per previous work,^33^ the solubility of the pure phases in the mixed solutions is estimated by straight line approximations from the pure component solubility (blue square for PHBA, pink square for β-GLU) through the corresponding invariant point into the cocrystal region. The solubility line for PHBA is shown in Figure 9 as a dashed red line and for β-GLU as a dashed green line. The diagram shows that in the experiments with solutions along the invariant point boundary lines, the solutions are not only supersaturated with respect to the cocrystal but also with respect to one of the pure solid phases, while in the experiments in stoichiometric solutions, the solution is only supersaturated with respect to the cocrystal.

As examples, illustrating the determination of the supersaturation with respect to the pure solid compounds, in Figure 9 the solid red line has been drawn from the PHBA apex through the most supersaturated solution point (open circle) on the E1 phase boundary line to intersect (black cross) with the solubility line for PHBA extrapolated into the CC region (where the pure PHBA solid is metastable) and the solid green line from the GLU apex through the most supersaturated solution point (open square) on the E2 phase boundary line to intersect (black cross) with the solubility curve for GLU extrapolated into the CC region (where the pure GLU solid is metastable). The composition at these intersection points represents the equilibrium solution composition for the respective metastable pure components in that system, and the invariant point represents the equilibrium composition for the 1:1 cocrystal. All experimental solution compositions on the E1 phase boundary line are supersaturated with respect to the cocrystal and with respect to pure solid PHBA but are undersaturated with respect to β-GLU. All experimental solution compositions on the E2 phase boundary line are supersaturated with respect to the cocrystal and β-GLU but are undersaturated with respect to PHBA. The supersaturation for each respective phase in each experiment along the phase boundary lines is presented in Table 6.

**Table 6:** Supersaturation, *S*, PHBA:GLU is the Supersaturation with Respect to the Cocrystal in the Non-Stoichiometric Nucleation Experiments^a^

E1^b^				E2^b^			
*S* PHBA:GLU	*X*^c^ PHBA	X*^d^ PHBA	*S* PHBA	*S* PHBA:GLU	*X*^c^ GLU	X*^d^ GLU	*S* GLU
**1.51**	0.01679	0.01535	**1.09**	**1.36**	0.03304	0.03296	**1.0025**
**1.64**	0.01730	0.01549	**1.12**	**1.40**	0.03320	0.03311	**1.0027**
**1.72**	0.01761	0.01557	**1.13**	**1.45**	0.03337	0.03327	**1.0029**
**1.94**	0.01847	0.01580	**1.17**	**1.69**	0.03430	0.03414	**1.0046**
**2.27**	0.01963	0.01611	**1.22**	**1.86**	0.03497	0.03477	**1.0058**
				**1.93**	0.03523	0.03501	**1.0063**

a Corresponding supersaturation with respect to the respective pure component is shown as *S* PHBA in E1 and *S* β-GLU in E2.

b E1 and E2 and refer to the nucleation experiments performed with solution compositions along the E1 and E2 phase boundary lines.

c *X* is the mole fraction concentration of pure PHBA or GLU at each of these points.

d X* is the equilibrium concentration of the pure component from the intersection point on the extrapolated solubility curve.

The nucleating solid in both non-stoichiometric systems is the pure PHBA:GLU cocrystal in all instances. As shown in Table 6, the supersaturation with respect to the cocrystal in the non-stoichiometric systems is in all experiments higher than the supersaturation with respect to the respective pure components, which of course, partly explains why the cocrystal is the nucleating solid. However, this greater supersaturation alone does not decide the nucleating phase as previously observed in the theophylline:glutaric acid (THP:GLU) cocrystal system. From a stoichiometric solution of THP:GLU, the pure solid-phase β-GLU is nucleating first as a metastable phase, which subsequently transforms into the stable cocrystal phase, even though the supersaturation with respect to the cocrystal is greater than the supersaturation with respect to β-GLU.^33^ The supersaturation with respect to the THP:GLU cocrystal ranged from *S* = 6.98 to 13.97, and the supersaturation with respect to the pure component β-GLU in the same system was from *S* = 1.179 to 1.240. The supersaturation with respect to β-GLU in the present work in the non-stoichiometric E2 system is much lower, Table 6, which can be part of the explanation for why the PHBA:GLU cocrystal is nucleating and not the β-GLU solid.

In order to further investigate why it is the PHBA:GLU cocrystal that nucleates instead of PHBA from the non-stoichiometric solutions in the E1 system and instead of β-GLU in the E2 system, *A* has been estimated for the pure compounds in these systems according to eqs 6 and 7. The calculations were made by assuming that the interfacial energy for nucleating the pure solids from the mixed solutions is the same as that valid for nucleating from the pure solutions, i.e., 1.52 mJ m^–2^ for PHBA and 2.43 mJ m^–2^ for β-GLU and the molecular volumes, υ_o_, and υ_MT_, are taken as equal and are those of the pure compounds. The *C*_e_ values used were obtained from the intersection points on the pure component solubility lines in the TPD (Figure 9) for each respective experiment; see the SI.

The estimated *A* value for PHBA in the E1 system for volume-diffusion-controlled nucleation ranges from 5.02 to 12.20 × 10^10^ m^–3^ s^–1^ and for interface-transfer-controlled is 10.10 to 10.63 × 10^10^ m^–3^ s^–1^. The *A* values for volume-diffusion control are slightly higher than those calculated for the nucleation of the cocrystal in the E1 system (Table 5). The interface-transfer controlled values are higher than those for the E1 system due to the higher *C*_e_ values. For GLU nucleation in the E2 system, *A* for volume-diffusion-controlled nucleation ranged from 0.07 to 0.30 × 10^10^ m^–3^ s^–1^, which is much lower than the corresponding *A* values for cocrystal nucleation in the E2 system, and for interface-transfer-controlled was 7.92 to 13.17 × 10^10^ m^–3^ s^–1^, which is higher than the corresponding *A* value for cocrystal nucleation in the E2 system due to the higher *C*_e_ values. Notably, the values for β-GLU nucleation in the E2 system are far below the *A* values obtained for nucleation of solid β-GLU from a pure solution as a result of the lower *C*_e_ values. The *A* values for PHBA nucleating from the E1 system are similar to the values for PHBA nucleation in a pure system, Table 5. According to these calculations, the kinetic advantage for pure component nucleation in the present system is far less obvious compared to in the THP:GLU system, where the calculated pre-exponential factor for β-GLU nucleation in a mixed solution is much higher than that for the THP:SA cocrystal, explaining the fact that β-GLU nucleated first.^33^

### Kinetics of Nucleation

**Figure 10:**
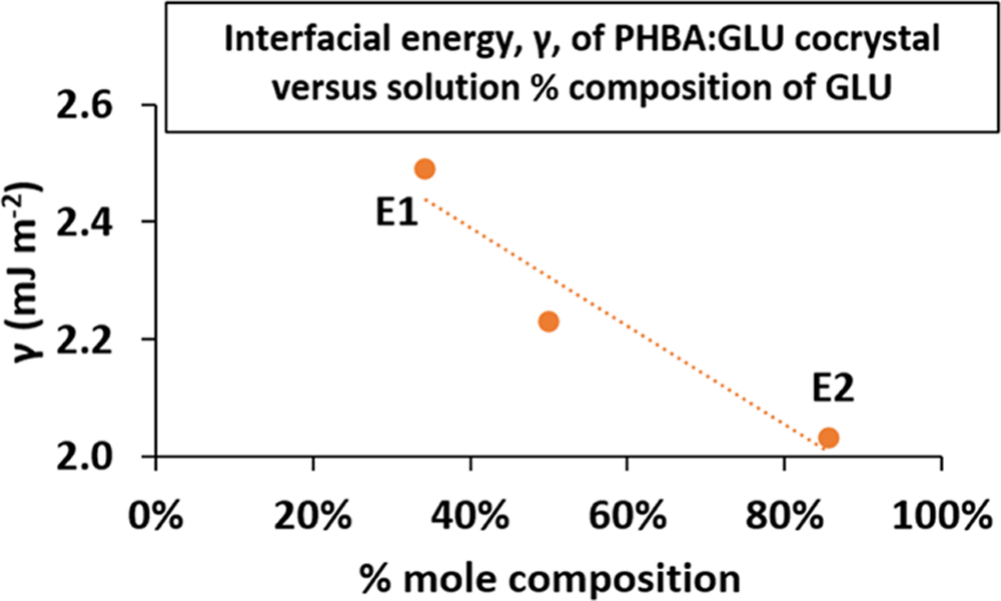
PHBA:GLU cocrystal interfacial energy versus the % composition of the solution in the mole fraction of GLU.

The interfacial energy of the PHBA:GLU cocrystal in a stoichiometric solution, 2.23 mJ m^–2^, is between the interfacial energy of the coformers:solid PHBA:γ = 1.52 mJ m^–2^ and β-GLU:γ = 2.43 mJ m^–2^ (Figure 10) and this is similar to what was observed in the THP:SA cocrystal system.^13^ The interfacial energy of the solid cocrystal nucleus changes somewhat with the solution composition, showing an inverse proportionality to the concentration of GLU in the mixed solution (Figure 9). The interfacial energy is the free energy difference between molecules at the surface of the crystal and molecules in the bulk of the crystal. Since the solid phase nucleating from the stoichiometric and non-stoichiometric solutions is the PHBA:GLU 1:1 cocrystal, differences in interfacial energy, as a first approximation, have to relate to the differences in the composition of the surrounding liquid phase. However, since the interfacial energy determined is an average value of the whole nucleus, this conclusion is not necessarily correct if the shape of the nucleus is very different in the three cases.

From the pure solutions, PHBA nucleates easier than β-GLU (Figure 4), and this is captured by a higher experimentally determined pre-exponential factor of 153 m^–3^ s^–1^ for PHBA versus 126 m^–3^ s^–1^ for β-GLU and a lower interfacial energy. When *A* is calculated according to the theoretical expressions, however, the *A* value for GLU is higher than for PHBA, irrespective of the mechanism, mainly related to the fact that the equilibrium concentration is higher for β-GLU. A possible explanation for this discrepancy is that the theoretical expressions are based on the fact that the transport rate is the governing factor, while in reality, conformational changes and specific desolvation effects can be important. In addition, GLU has been reported to form cyclic dimers in solution with aprotic solvents, and the effect of dimerization on the calculations of *A* is discussed in the SI.

### Is Nucleation of the Cocrystal More Difficult?

**Figure 11:**
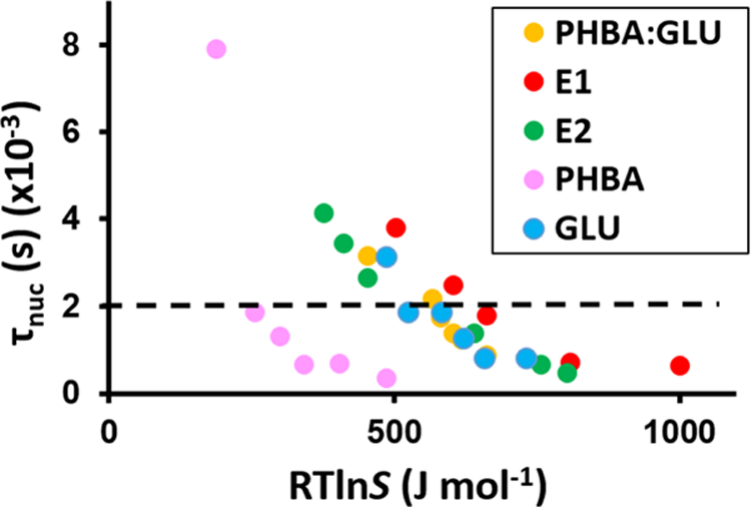
Nucleation time versus *RT* ln *S*, using *S* as per eq 9, for the PHBA:GLU cocrystal systems.

As stated above, one of the conclusions of the present work is that the nucleation of the cocrystal is more difficult than the nucleation of the respective pure components. The basis for this statement is that to reach the same induction time, a higher driving force is required for the cocrystal. The experiments are designed to deliver an induction time in a convenient time span: not too short such that the time of cooling to reach *T*_nuc_ would not be negligible, and not too long because that makes the experimental work more time-consuming and practically inconvenient. For each system, the solution concentrations are adjusted to deliver an induction time within the desired time span. For a pure system, the definition of supersaturation is straightforward. However, for the cocrystal system, this is partly a matter of choice. The supersaturation driving force, *kT* ln *S*, characterizes the free energy difference between the solution state and the crystalline state. Above, this has been defined per heterodimer assembly, making up the cocrystal crystalline structure (structure in the SI), in accordance with eq 2. However, this leads to the conclusion that there is a difference in “dimensionality” in the definition of *S* for the pure systems (eq 1) compared to the definition of S for the cocrystal systems (eq 2). Of course, in both equations, *S* is dimensionless, but for the cocrystal system, *S* receives units that are mole fractions squared in the nominator and denominator, while for the pure systems, it is a ratio of just mole fractions. When the definition of *S* according to eq 2 is inserted into the expression *kT* ln *S*, it represents the free energy change per cocrystal heterodimer because the underlying reaction of change forms one heterodimer of the solid state. However, if it is defined as the free energy change per reactant molecule, *S* is defined as per eq 9
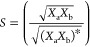
9which, for a stoichiometric 1:1 cocrystal, gives ln *S* half of the value obtained using eq 2. For a driving force comparison with the pure systems, this actually appears to be a more appropriate definition because it gives the same dimensionality for the pure and the cocrystal systems. Using eq 9 for the 1:1 cocrystal to be compared with the values for the pure systems, according to eq 1, it turns out, as shown in Figure 11, that the cocrystal is no longer more difficult to nucleate than the pure compounds but is approximately equally difficult as the more difficult-to-nucleate pure compound, i.e., β-GLU.

**Figure 12:**
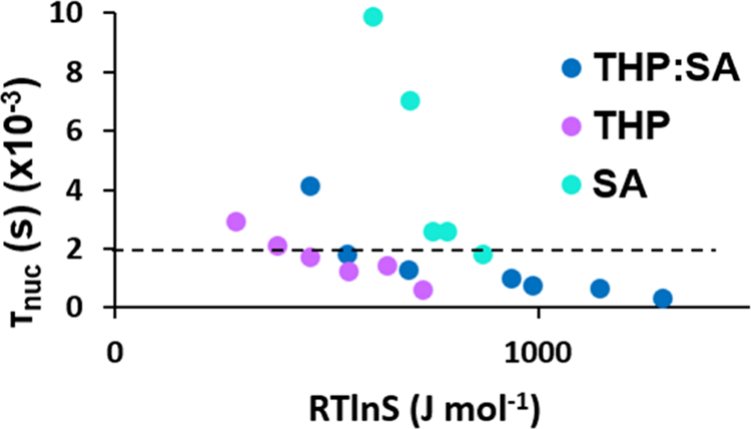
Nucleation time versus *RT* ln *S* using the new value for *S* for the THP:SA cocrystal system.

In the previous work on the THP:SA cocrystal, it was also concluded that the nucleation of the cocrystal was more difficult than the nucleation of the pure compounds.^13^ However, if eq 9 is adopted to characterize the driving force for nucleation of the THP:SA cocrystal, it is found that this statement is no longer correct (Figure 12).

For example, for an induction time of 2000 s, the driving force according to the definition given in eq 9 for nucleation of the cocrystal is higher than the driving force for nucleation of THP but lower than that for nucleation of SA, i.e., the nucleation difficulty of the THP:SA cocrystal now falls in between the nucleation difficulties of the two pure compounds. Overall, the diagram shows that at high supersaturation, the cocrystal is about equally difficult to nucleate as pure SA, while at lower supersaturation, it comes closer to the behavior of THP. The *S* values of the exponential term defined by eq 9 for both the PHBA:GLU and THP:SA cocrystal systems are detailed in the SI.

If the same consideration is applied in the evaluation of the nucleation data, we need to go back to the original derivation of the CNT equation. The derivation starts with a free energy balance for one nucleus bringing molecules from the supersaturated solution into the crystalline state. The change in free energy is just the volume of the nucleus times the free energy difference between the solution state and the crystalline state per unit volume of the nucleus, *g*_v_. We add a correction term to the equation to account for the fact that the molecules at the surface of the nucleus have a higher free energy than the molecules fully inside the crystal structure, i.e., we subtract a term which is the surface area times the interfacial energy. In the conversion of *g*_v_ into free energy difference per molecule, i.e., *kT* ln *S*, we introduce the molecular volume, υ, as given by eq 10
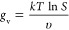
10Defining *S* as per eq 9, i.e., per reactant molecule, requires the molecular volume in eq 10, υ, to be half of that of the PHBA:GLU heterodimer assembly. As a consequence, the exponential term in eq 5 does not depend on how the supersaturation is defined for the cocrystal since the changes in the driving force will be balanced out by the changes in the molecular volume, and accordingly, the interfacial energy determined from the experimental data remains the same. However, the slope of the CNT graph will change, and along with that, the experimentally determined pre-exponential factor, thus leading to changes in the order of the difficulty of nucleation. If the nucleation time was compared at equal *g*_v_, the outcome would be the same as that of using eq 9 for the cocrystal.

## Conclusions

Nucleation experiments of the PHBA:GLU cocrystal from a stoichiometric solution and PHBA and β-GLU from the respectively pure solutions revealed that in terms of nucleation difficulty, pure PHBA required the lowest driving force for the same induction time, followed by β-GLU, and the PHBA:GLU cocrystal was the most difficult to nucleate. This relation is captured by the values determined for the interfacial energy and pre-exponential factor where the cocrystal had the lowest pre-exponential factor and intermediate interfacial energy, β-GLU had the highest interfacial energy and intermediate pre-exponential factor, and PHBA had the lowest interfacial energy and the highest pre-exponential factor. The pre-exponential factors determined from theoretical expressions for volume-diffusion and interface-transfer controlled nucleation mechanisms agreed with the order of the experimentally determined pre-exponential values except for GLU, which theoretically had a relatively higher value than experimentally determined, possibly due to dimerization in solution. The nucleation from non-stochiometric solutions with compositions along the invariant point boundary lines revealed that although pure PHBA and β-GLU are also stable solid forms in these systems, it is always the pure PHBA:GLU cocrystal that is nucleating. The nucleation difficulty of the cocrystal from stoichiometric and non-stoichiometric solutions was quite similar. If the driving force for nucleation of the cocrystal is defined per reactant molecule instead of per heterodimer, the cocrystal nucleation difficulty is close to that of the more difficult-to-nucleate pure compound.

## Supporting Information Available

Solid-phase characterization techniques, PXRD patterns, DSC curves, SEM micrographs, solubility values, nucleation parameter values, and derivation of pre-exponential factor equations (PDF)
