# Supplementary material for: Investigation into the Nucleation of the p-Hydroxybenzoic Acid:Glutaric Acid 1:1 Cocrystal from Stoichiometric and Non-Stoichiometric Solutions
Source: Cryst Growth Des. 2023 Sep 1;23(10):7053–65. doi: 10.1021/acs.cgd.2c01522 (PMC10557069; doi:10.1021/acs.cgd.2c01522)
Supplement: Supplementary file 1 — cg2c01522_si_001.pdf [file cg2c01522_si_001.pdf]

Investigation into the nucleation of the *p*-hydroxybenzoic acid:glutaric acid 1:1 cocrystal from stoichiometric and non-stoichiometric solutions

Hannah McTague[1] and Åke C. Rasmuson*[1,2]

[1]*Synthesis and Solid State Pharmaceutical Centre (SSPC), Bernal Institute, Department of Chemical and Environmental Science, University of Limerick. Limerick V94 T9PX, Ireland.*
[2]*Department of Chemical Engineering and Technology, KTH Royal Institute of Technology, SE-100 44 Stockholm, Sweden.*

Characterisation

<u>Solubility determination</u>

The solubility of the stochiometric 1:1 co-crystal was measured by dissolving PHBA:GLU 1:1 cocrystal solid in excess in acetonitrile and allowing equilibration for 72 hours. The samples were then allowed to rest and the solid to settle for an hour. Using heated needles and syringes a sample of the liquid phase was obtained and filtered through heated 0.2 µm PTFE filters into a pre-weighed glass vial and sealed immediately. The weight was then taken of the sample and vial. The samples were evaporated to dryness over one week in a protected environment in a fume hood. The dry samples were then weighed and then weighed again 24 hours later to ensure evaporation was complete. The solubility was then calculated in g/g from the difference in the sample weight before and after evaporation of the solvent. The excess solid phase was analysed after equilibration and analysed to ensure it was pure PHBA:GLU cocrystal.

<u>PXRD</u>

PXRD data were collected in reflectance mode with an Empyrean diffractometer (PANalytical, Phillips) equipped with CuK$\alpha_{1,2}$ radiation (γ = 1.5406 Å) operating at 40 kV and 40 mA at room temperature. Samples were scanned between 2θ values of 5 and 40° at a step size of 0.01313° 2θ/s, 73 s per step.

<u>DSC</u>

Differential scanning calorimetry (DSC) was performed on a Netsch Polyma 214 DSC. Samples were analysed in a nitrogenous environment with a temperature ramp rate of 10 °C min^{-1} over a temperature scan range from 30 °C and 230 °C. Crystals were isolated from solvent with Whatman filter paper. 5-7 mg of crystals were added to concavus aluminium pans which were sealed using a crimping press and then the lid was pierced. The instrument was calibrated using samples of indium and lead.

<u>SEM</u>

For Scanning Electron microscopy (SEM), sample analysis was carried on the Jeol CARRYSCOPE, coated in gold by a 45-second sputter to ensure a fine coating and minimise ionization.

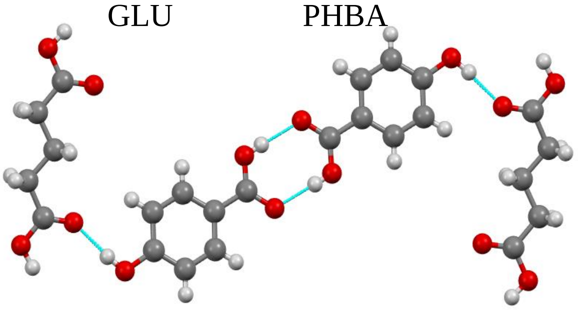

Figure 1 PHBA:GLU 1:1 cocrystal.

The unit cell of PHBA:GLU 1:1 cocrystal, **Figure 1**, has a $R_2^2(8)$ carboxylic acid homodimer present between each molecule of PHBA, with each dimer capped at both ends through an OH···C=O hydrogen bond of PHBA and GLU, respectively.[1]

The mol L^{-1} concentration,

Table 1 is calculated using the molecular mass of MeCN, 41.05 g mol^{-1} and the density of pure MeCN at 20 °C of 782 kg m^{-3}. The molecular mass of PHBA:GLU is 270.24 g mol^{-1} representing the 1:1 dimer of PHBA and β-GLU.

Table 1 solubility in g g^{-1} and mol L^{-1} for PHBA:GLU cocrystal and pure PHBA and GLU.

**g g^{-1} in MeCN**			
**°C**	PHBA	GLU	PHBA:GLU
**2**	0.0266[a]	0.0384[a]	-
**10**	-	-	0.0459
**20**	0.0433[a]	0.0989[a]	0.0700
**40**	0.0757[a]	0.3534[a]	0.1735
**mol L^{-1}**			
**°C**	PHBA	GLU	PHBA:GLU
**20**	0.2454	0.5858	0.2025

[a] data from Yang *et. al*[1]

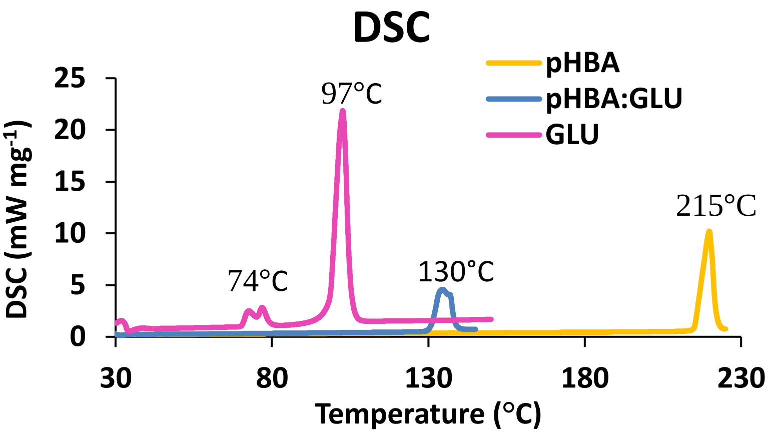

Figure 2 DSC data from PHBA:GLU cocrystal versus pure PHBA and GLU.

Table 2 Molecular weights and melting points for PHBA, β-GLU and PHBA:GLU 1:1 cocrystal.

	Mr	Melting point
**PHBA**	138.12	213-215 °C[1,2]
**β-GLU**	132.12	β-α at 74, 97 °C
**PHBA:GLU**	270.24	133 °C

Samples from induction time experiments and cocrystal synthesis slurry were taken and analysed by PXRD. The PHBA:GLU cocrystal undergoes two endothermic events during melting with the onset of the first peak at 130 °C and another at 136 °C, **Figure 7**.

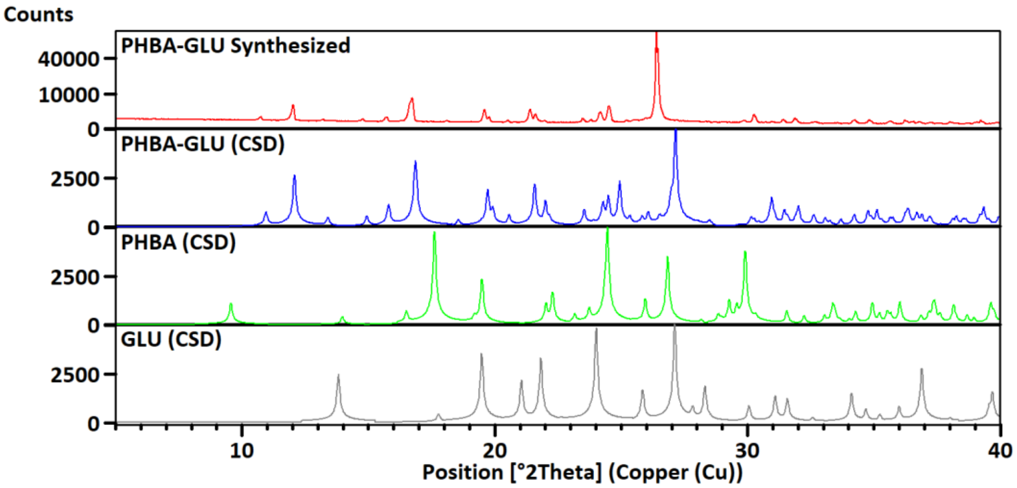

Figure 3 PXRD diffractogram of the PHBA:GLU cocrystal after synthesis.

The samples taken from the slurry were proved to be PHBA:GLU cocrystal. Although from the PXRD image in **Figure 3** above, the pattern of PHBA:GLU experimental is different to the theoretically calculated pattern in the CSD, however, this aligns with findings by the discoverers of the cocrystal.[1]

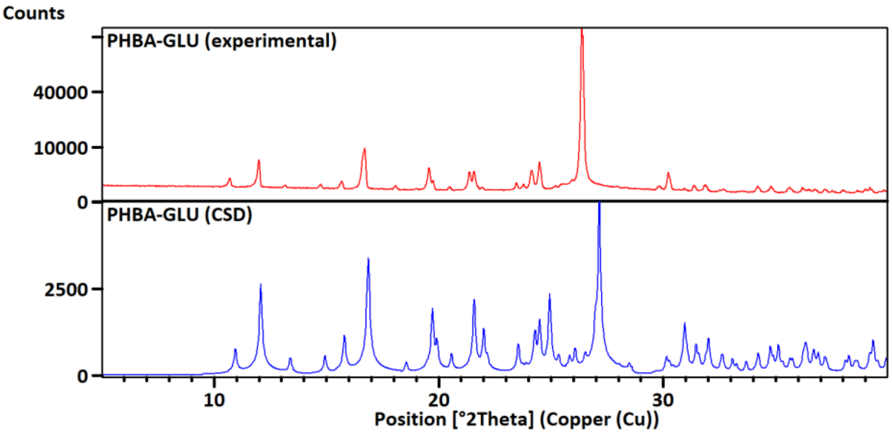

Figure 4 PXRD diffractogram of PHBA:GLU as synthesized is different to the theoretically predicted pattern. The diffractogram of PHBA:GLU experimental aligns with the pattern of PHBA:GLU 1:1 cocrystal obtained experimentally by the discoverers of the cocrystal.[1]

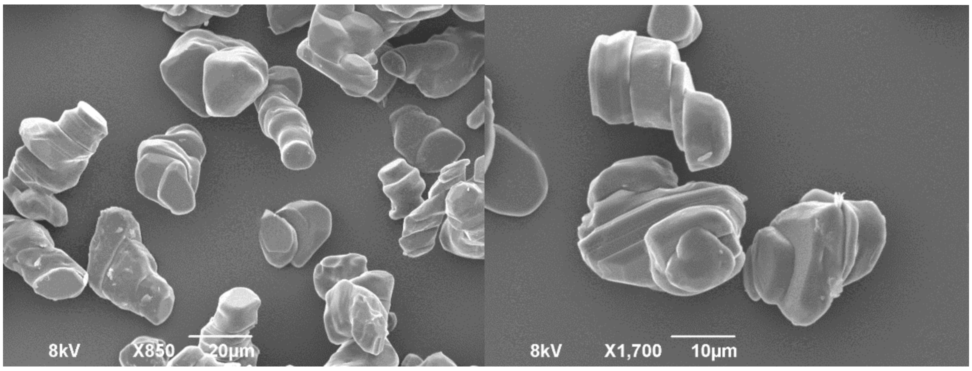

Figure 5 SEM taken of PHBA:GLU after synthesis.

SEM images taken of samples from synthesis batches show the cocrystal in a stacked arrangement of rounded blocks.

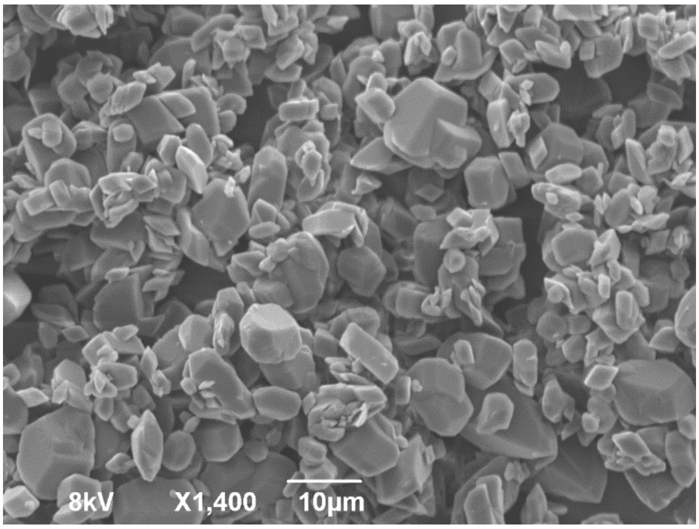

Figure 6 SEM of filtrate from PHBA:GLU 1:1 solution, S=1.61.

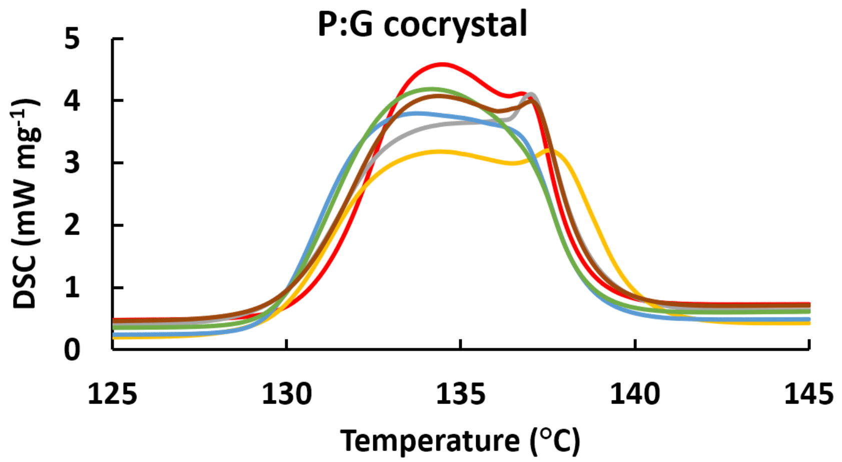

Figure 7 DSC profile of the PHBA:GLU cocrystal crystallised from a 1:1 solution over a range of supersaturations.

Any differences in the DSC outputs for the cocrystal can be attributed to differences in the sample size. All samples taken from PHBA:GLU induction time experiments and also from cocrystal synthesis slurries showed a similar DSC pattern and thermal event at 136 °C after the onset of melting at 130 °C. All samples showed diffractograms identical to the PHBA:GLU 1:1 cocrystal.

Table 3 Cocrystal region width (mole fractions) from the TPDs provided by Yang *et al*. [1]

**2 °C in acetonitrile**				**Width**
**Solid phase**	PHBA	GLU	MeCN	3D distance
**PHBA and CC**	0.00804	0.00409	0.988	0.010834
**GLU and CC**	0.00282	0.0127	0.984	
**20 °C in acetonitrile**				
**Solid phase**	PHBA	GLU	MeCN	
**PHBA and CC**	0.0147	0.00768	0.978	0.027567
**GLU and CC**	0.00533	0.0316	0.968	
**40 °C in acetonitrile**				
**Solid phase**	PHBA	GLU	MeCN	
**PHBA and CC**	0.0299	0.0218	0.948	0.123805
**GLU and CC**	0.0153	0.116	0.869	

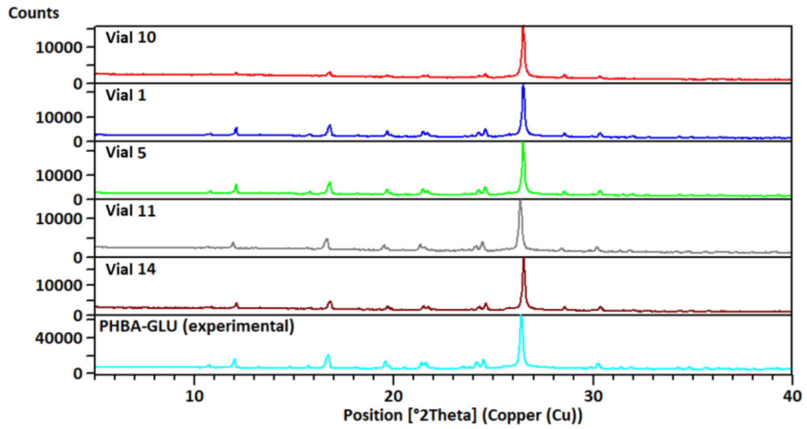

Figure 8 PXRD patterns of the solid residue from samples filtered from PHBA:GLU induction time experiments in stoichiometric solutions. The pattern for pure PHBA:GLU cocrystal is also shown.

In **Figure 8** the samples were taken at time points ranging from 30 seconds up to 8 hours after the first signs of nucleation, **Table 4**. In all cases the solid residue was identified as pure PHBA:GLU cocrystal supporting that the cocrystal is the nucleating solid and remains as the stable solid phase.

Table 4 Sample details for Figure 8 where *S* is the supersaturation of the batch from which samples were taken and the time refers to the time in seconds between the first signs of nucleation and filtration of the sample for analysis.

Vial no.	S	Time (s)
**10**	1.66	28800
**1**	1.72	600
**5**	1.72	90
**11**	1.72	30
**14**	1.72	300

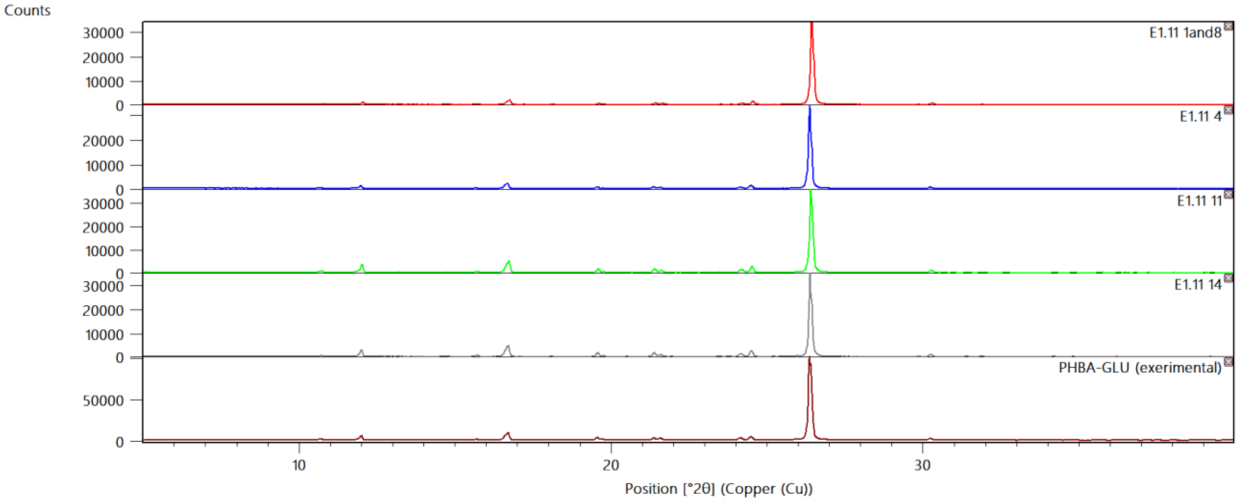

Figure 9 E1.11 (S=1.93) filtered at different time points from 1 minute after nucleation up to 17 minutes after nucleation. All PXRDs identify pure PHBA:GLU 1:1 cocrystal as the solid.

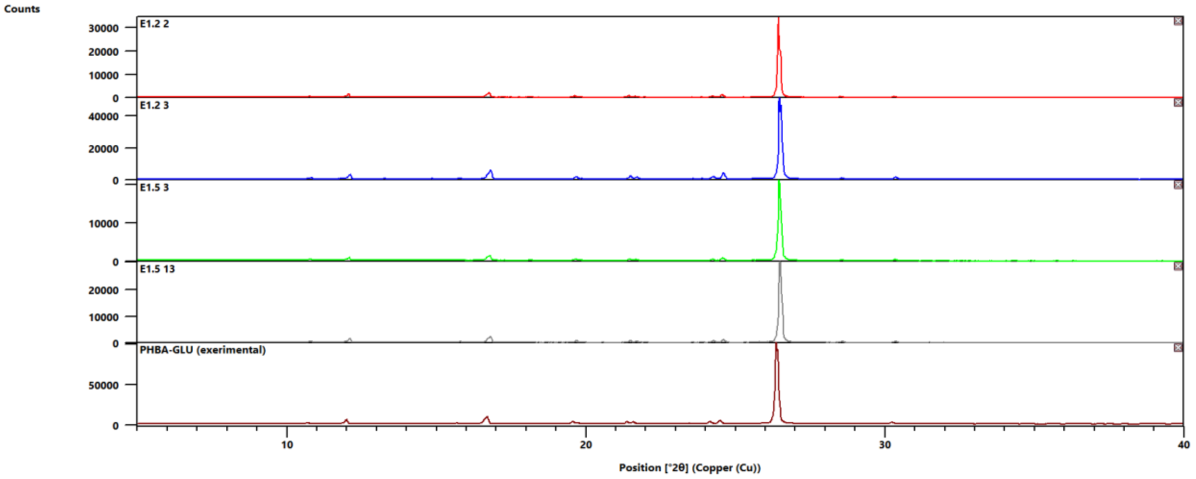

Figure 10 PXRDs from nucleation experiments in non-stoichiometric solutions lean in GLU (E1 solutions, S=2.27), match pure PHBA:GLU cocrystal.

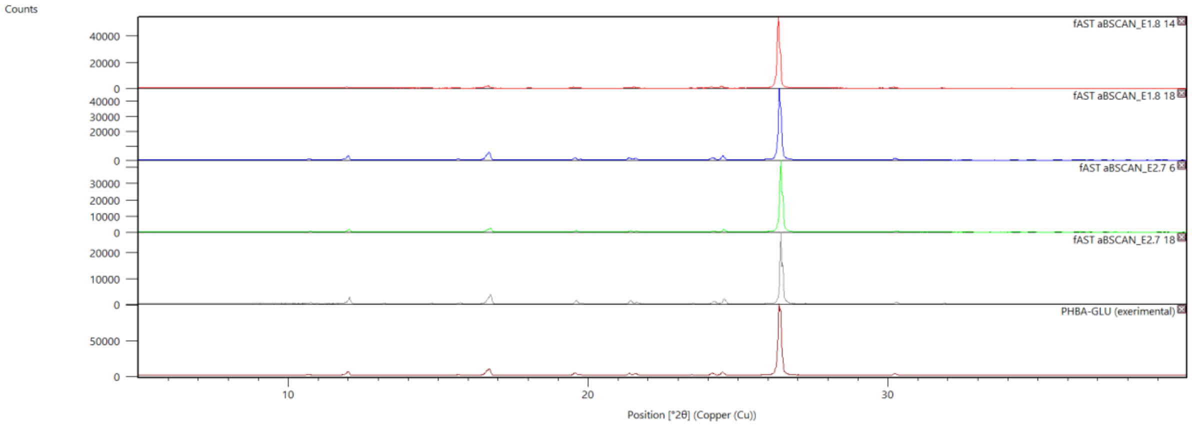

Figure 11 PXRDs from nucleation experiments in non-stoichiometric solutions lean in GLU (E1 solutions, S=1.72) and solutions lean in PHBA (E2 solutions, S=1.36) match pure PHBA:GLU cocrystal.

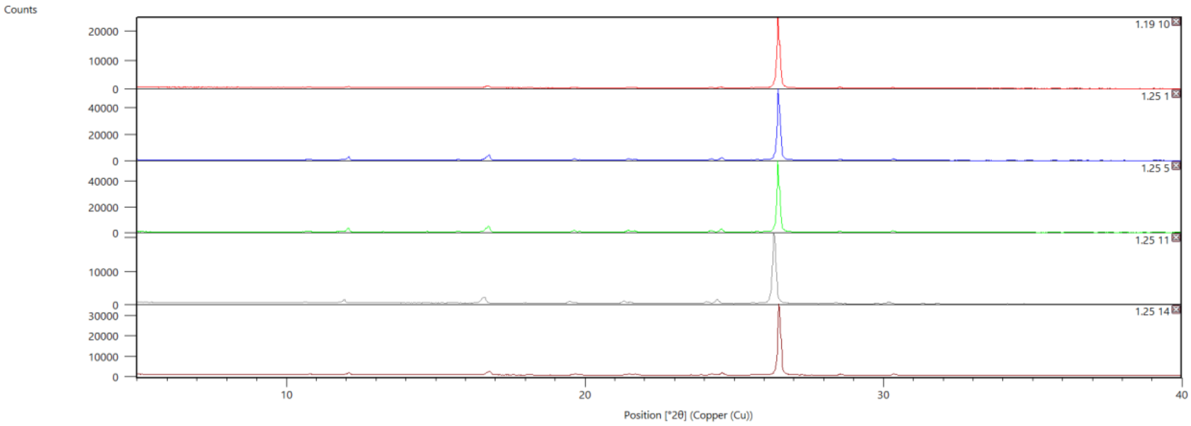

Figure 12 PXRDs from nucleation experiments in non-stoichiometric solutions lean in GLU (E1 solutions) and solutions lean in PHBA (E2 solutions) match pure PHBA:GLU cocrystal.

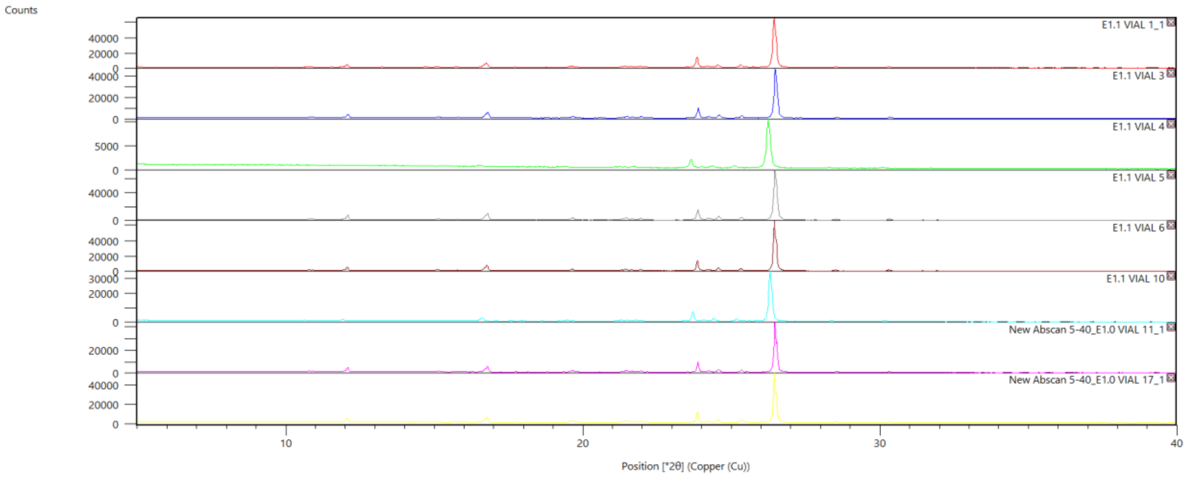

Figure 13 PXRDs from nucleation experiments in non-stoichiometric solutions lean in GLU (E1 solutions) match pure PHBA:GLU cocrystal.

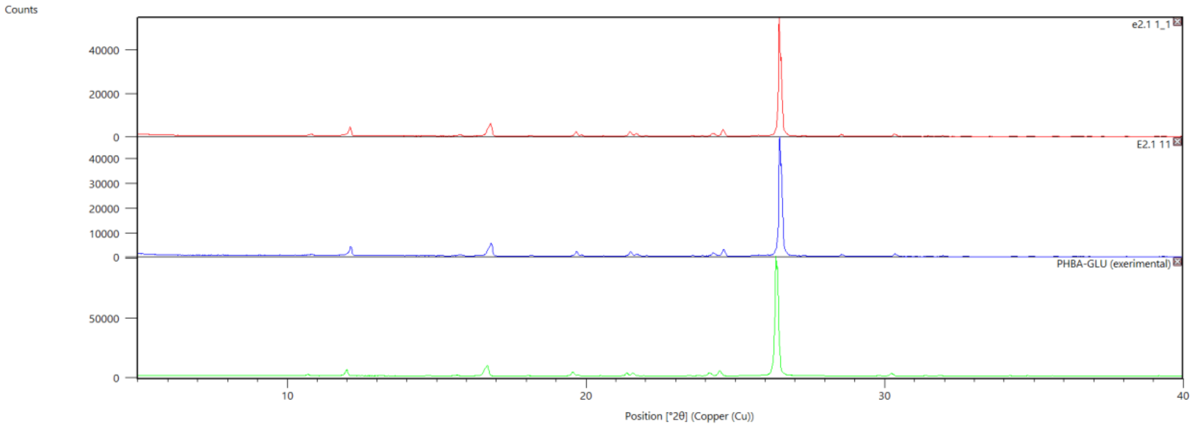

Figure 14 PXRDs from nucleation experiments in non-stoichiometric solutions lean in GLU (E1 solutions) and solutions lean in PHBA (E2 solutions S=1.93) match pure PHBA:GLU cocrystal.

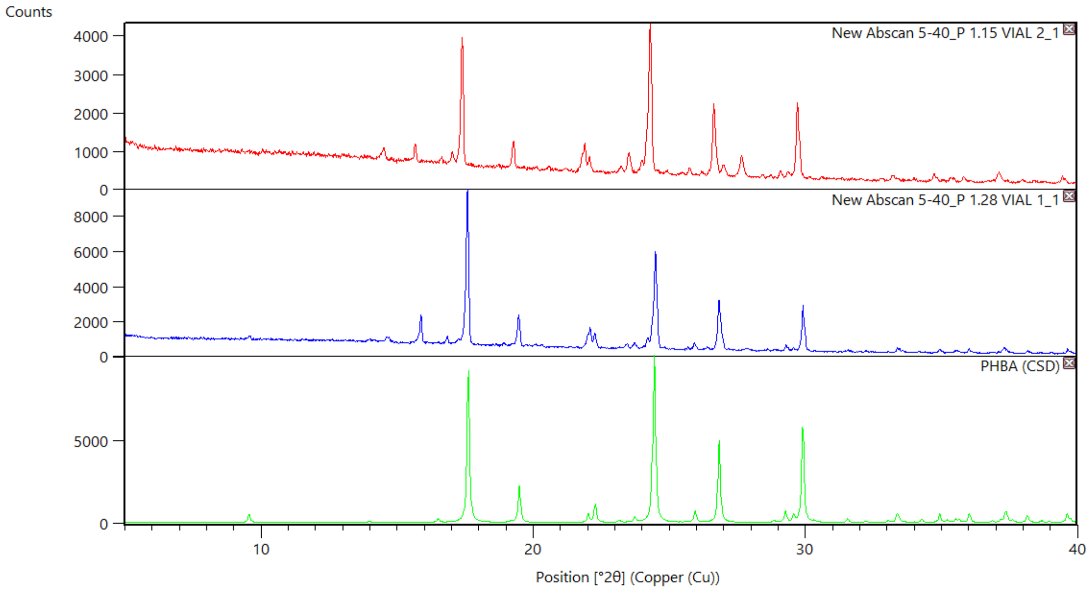

Figure 15 PXRDs from nucleation experiments in pure PHBA solutions (S= 1.15 and S= 1.22) show the solid phase is pure PHBA (CSD ref. code: JOZZIH01).

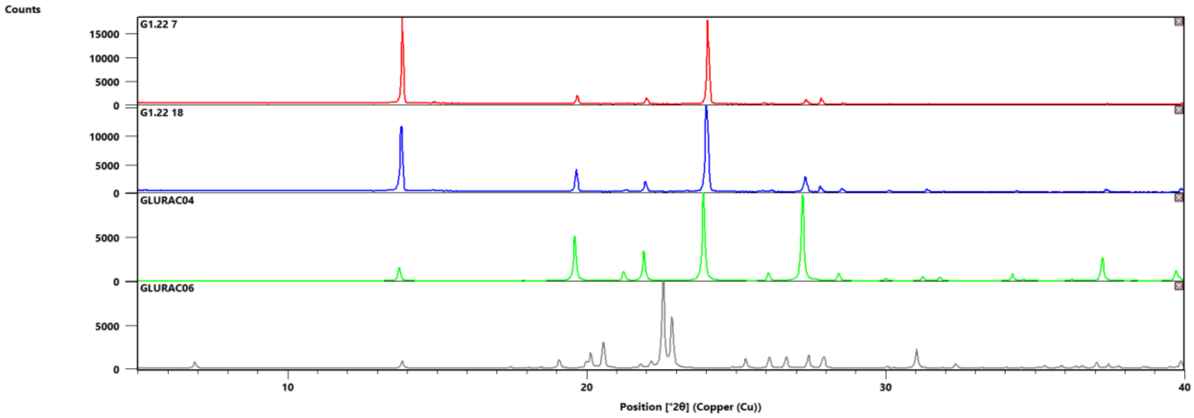

Figure 16 PXRDs from nucleation experiments in pure GLU solutions show the solid phase is pure β-glutaric acid (CSD ref. code: GLURAC04).

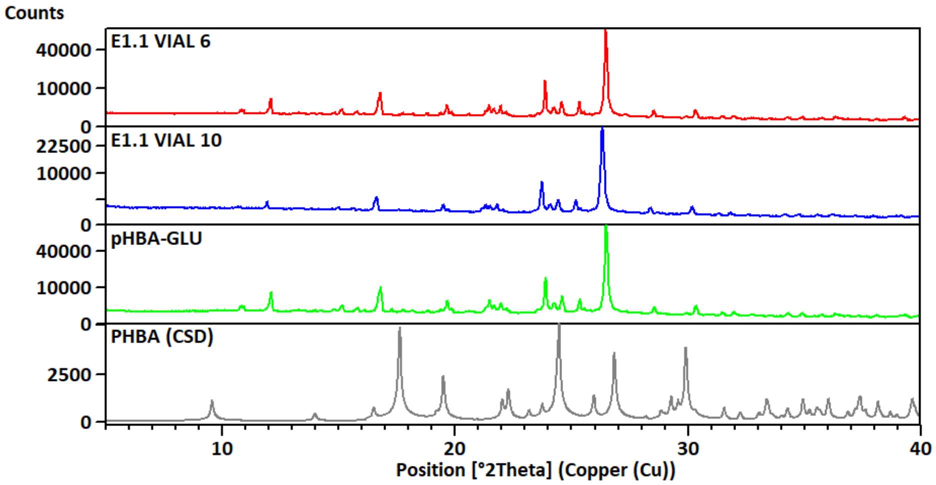

Figure 17 PXRDs from nucleation experiments in non-stoichiometric solutions lean in GLU (E1 solutions) and solutions lean in PHBA (E2 solutions) match pure PHBA:GLU cocrystal.

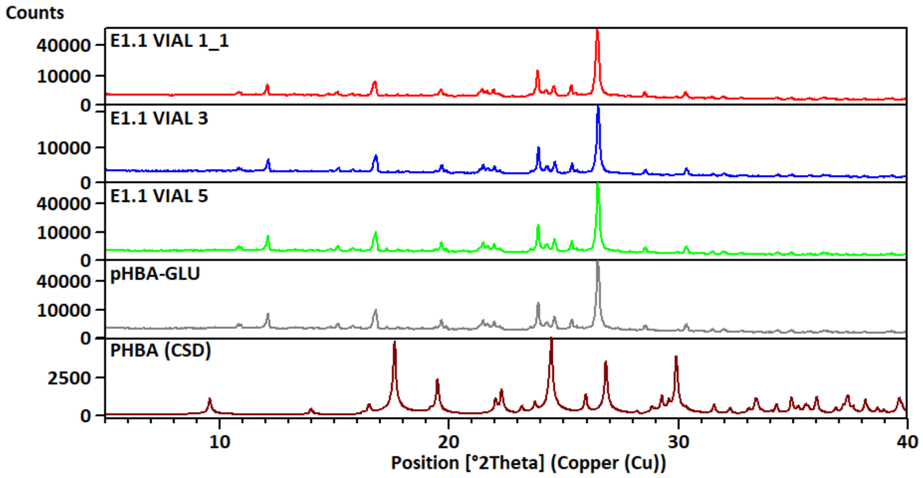

Figure 18 PXRDs from nucleation experiments in non-stoichiometric solutions lean in GLU (E1 solutions) and solutions lean in PHBA (E2 solutions) match pure PHBA:GLU cocrystal.

Nucleation

Probability Distributions

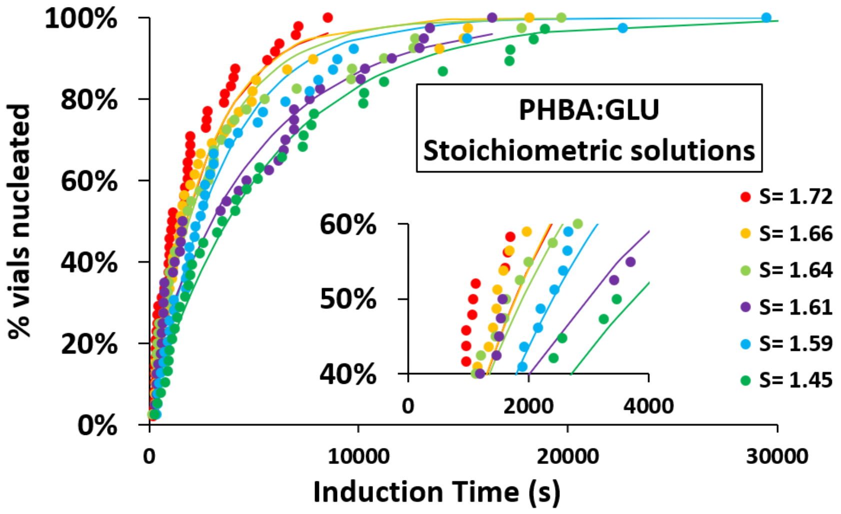

Figure 19 Induction time probability distributions, *P*(τ), for PHBA:GLU from stoichiometric solutions at 20 °C. The solid line is the Poisson distribution function fitted to the experimental data. Magnified image of *P*(τ) in lower right.

In Figure 19 the fitting curve for S=1.72 is close to the S= 1.66 curve, however, separation can be seen towards the end of the curve as the % vials nucleated is increased.

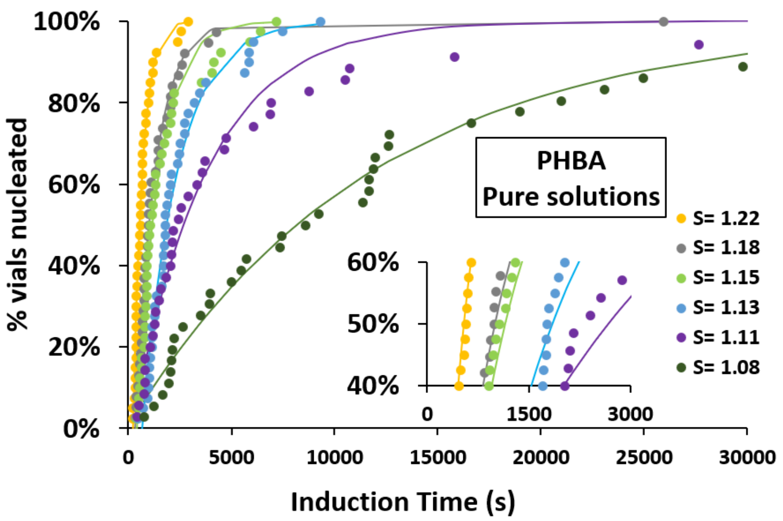

Figure 20 Induction time probability distributions, *P*(τ), for PHBA from a pure solution at 20 °C. The solid line is the Poisson distribution function fitted to the experimental data. Magnified image of *P*(τ) in lower right.

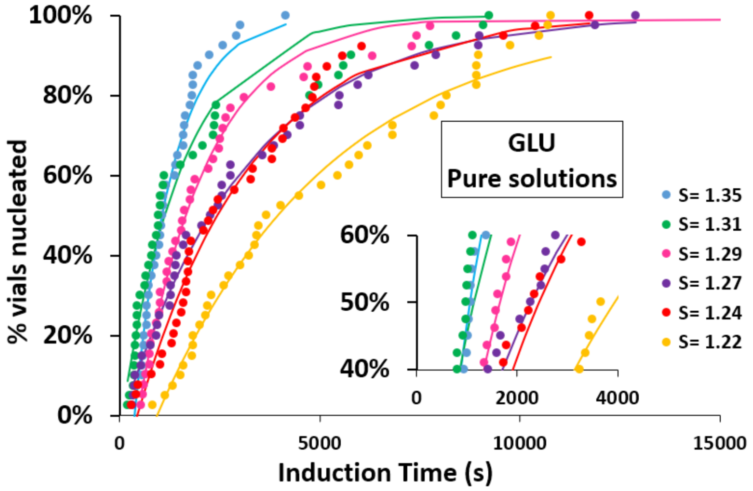

Figure 21 Induction time probability distributions, *P*(τ), for β-GLU from a pure solution at 20 °C. The solid line is the Poisson distribution function fitted to the experimental data. Magnified image of *P*(τ) in lower right.

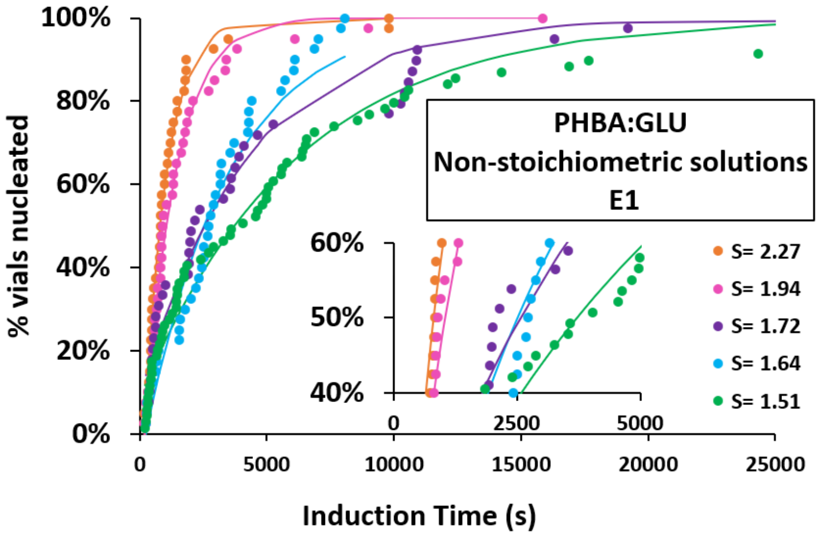

Figure 22 Induction time probability distributions, *P*(τ), for nucleation experiments at 20 °C performed along the E1 phase boundary at different supersaturation ratios (*S*) with respect to the cocrystal. The solid line is the Poisson distribution function fitted to the experimental data. Magnified image of *P*(τ) in lower right.

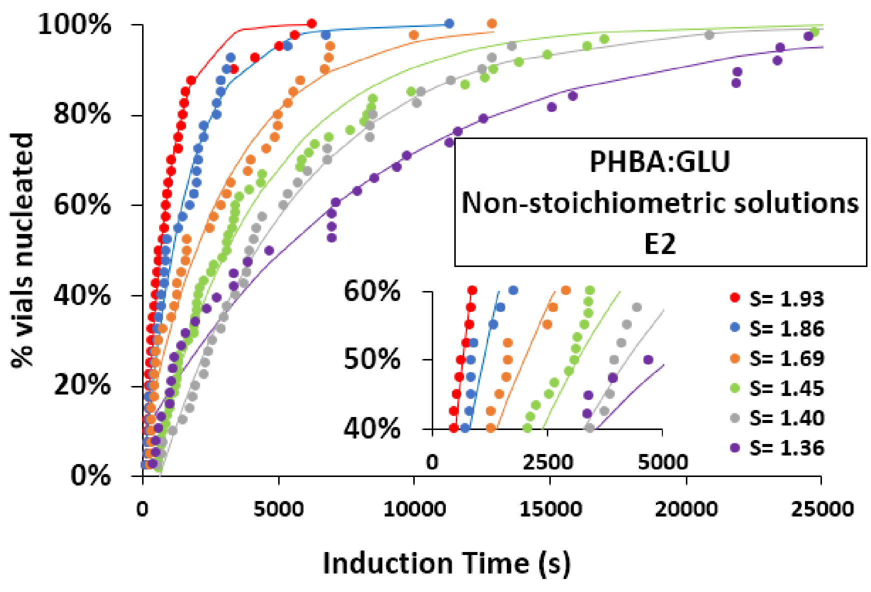

Figure 23 Induction time probability distributions, *P*(τ), for nucleation experiments at 20 °C performed along the E2 phase boundary at different supersaturation ratios (*S*) with respect to the cocrystal. The solid line is the Poisson distribution function fitted to the experimental data. Magnified image of *P*(τ) in lower right.

The fitted Poisson distribution function is used to calculate for each experimental point the corresponding value delivered by the Poisson function, and in the graph a straight line is drawn between the calculated points. The 'kinks' are can be observed in the upper sections of certain graphs where there is more of a spread in induction times.

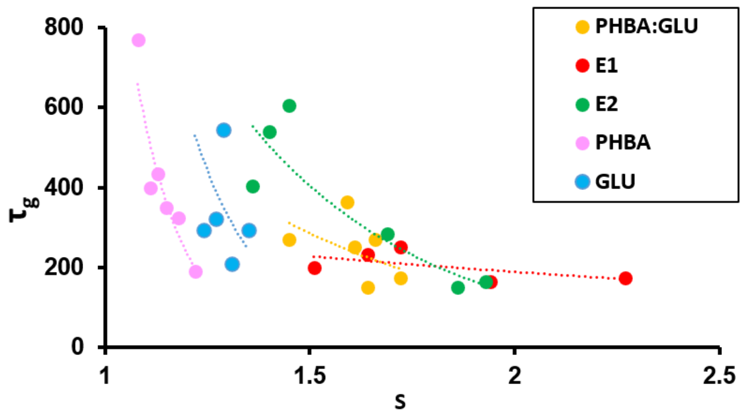

Figure 24 τ_g (growth time) dependence on *S*. The points are estimated from the experimental data and the lines are exponential fittings.

Table 5 Nucleation growth parameter extracted directly from the Poisson distribution Eq [3] of main manuscript. Negative τ_g values have been highlighted in red.

	τ_g Poisson			
**PG**	PHBA	GLU	E1	E2
**-223**	-46	925	-565	-811
**102**	184	440	233	655
**-762**	657	92	-389	253
**-178**	403	452	190	-191
**-2**	335	70	201	12
**-38**	223	369	-	79

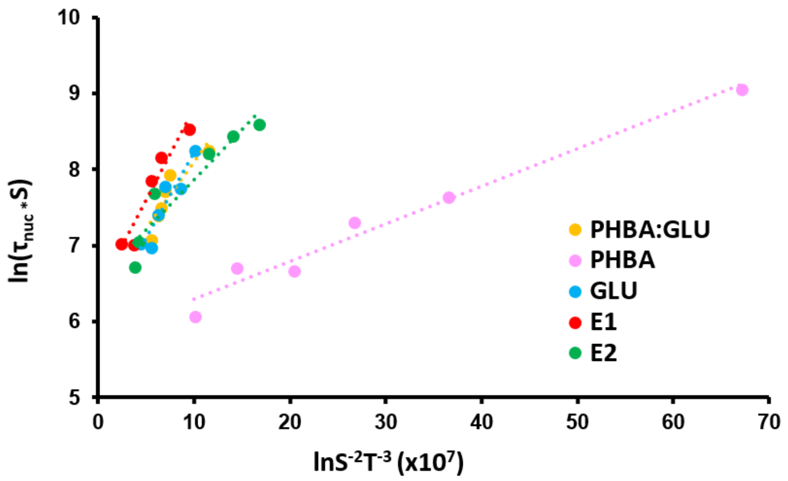

Figure 25 CNT plot for PHBA:GLU nucleation from stoichiometric, E1 solutions, E2 solutions, PHBA and β-GLU nucleation from pure solutions.

Table 6 Pre-exponential factor, *A*, calculated for the nucleation of PHBA and β-GLU from pure systems according to Eq [6] manuscript and Eq [7] manuscript. C_e is in mol m^{-3} and the *A* values are $\times 10^{10}$ m^{-3} s^{-1}.

**PHBA**			
**S**	Ce	A, Volume-diffusion	A, Interface-transfer
**1.08**	245	2.04	4.63
**1.11**	245	2.77	4.63
**1.13**	245	3.24	4.63
**1.15**	245	3.71	4.63
**1.18**	245	4.39	4.63
**1.22**	245	5.28	4.63
**β-GLU**			
**S**	Ce	A, Volume-diffusion	A, Interface-transfer
**1.22**	586	9.86	13.79
**1.24**	586	10.66	13.79
**1.27**	586	11.85	13.79
**1.29**	586	12.62	13.79
**1.31**	586	13.38	13.79
**1.35**	586	14.87	13.79

Table 7 Pre-exponential factor, *A*, calculated by hypothetically assuming pure component nucleation in the non-stoichiometric systems according to volume-diffusion controlled nucleation and interface-transfer controlled nucleation. C_e is in mol m^{-3} of the rate limiting component and the *A* values are $\times 10^{10}$ m^{-3} s^{-1}.

**PHBA nucleating in E1**			
**S**	Ce	A, Volume-diffusion	A, Interface-transfer
**1.09**	300	2.77	5.78
**1.12**	286	3.47	5.51
**1.13**	305	3.98	5.87
**1.17**	303	5.09	5.83
**1.22**	316	6.72	6.09
**GLU nucleating in E2**			
**S**	Ce	A, Volume-diffusion	A, Interface-transfer
**1.0025**	186	0.04	4.39
**1.0027**	195	0.04	4.60
**1.0029**	205	0.05	4.83
**1.0046**	257	0.10	6.06
**1.0058**	295	0.14	6.95
**1.0063**	309	0.16	7.30

THP:SA nucleation parameters using *S* in the y-coordinate of the CNT equation as per THP (limiting component)

The nucleation difficulty of the THP:SA cocrystal system from a previous study[3] using *S* as per **Eq [13]** is in between the two pure compound difficulties**.** The nucleation difficulty defined in this manner is perfectly captured by the new γ and *A* values obtained from the CNT plot in **Figure 26**.

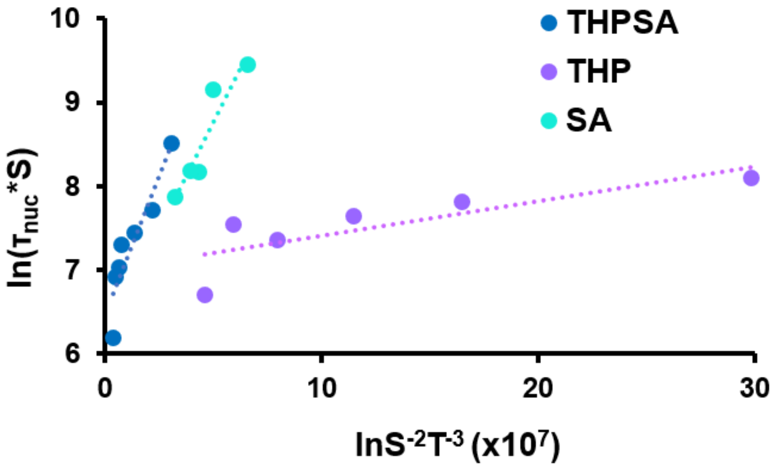

Figure 26 CNT plot using *S* as per limiting component in the y-coordinate and *S* as per THP:SA heterodimer in the exponential term.

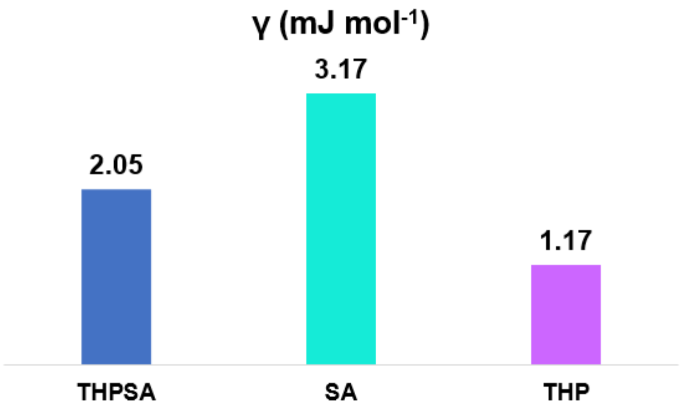

Figure 27 Interfacial energy for THP:SA system calculated from CNT plot, Figure 26.

The order of the new γ and *A* values, **Figure 27** and **Figure 28**, are the same as the original calculations[3] however the interfacial energy values have changed slightly and the pre-exponential factor has increased as expected with the reduced *S* in the y-coordinate by this definition.

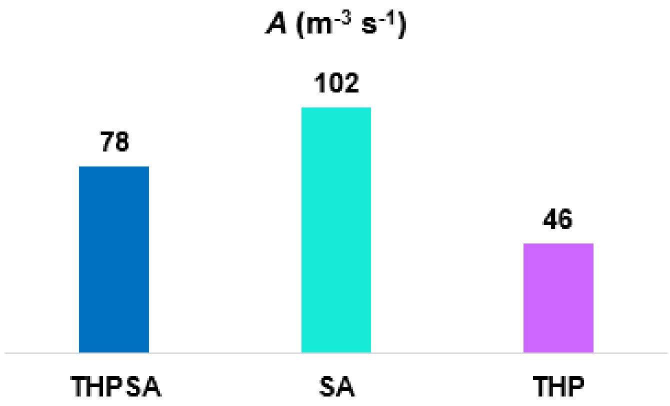

Figure 28 Pre-exponential factor for THP:SA system calculated from CNT plot, Figure 26.

Derivation of Eqs [6] and [7] from main manuscript

The nucleation rate, J, is given as **Eq [1]**[4]:

$$J = zf^*C^* \quad \textbf{[1]}$$

The equilibrium concentration of nuclei C^* is given by **Eq [2]**[4]:

$$C^* = C_o \exp\left(-\frac{W^*}{kT}\right) \quad \textbf{[2]}$$

The pre-exponential factor, A, is given by **Eq [3]**:

$$A = z\frac{f^*}{S}C_o \quad \textbf{[3]}$$

C_o is the concentration of nucleation sites in the solution. All molecules are assumed to be able to act as nucleation sites and since the solvent molecules are the most abundant and for simplicity C_o is taken as the inverse of the solvent molecular volume, V_o (m^3)[4,5]:

$$C_o = \frac{1}{V_o} \quad \textbf{[4]}$$

For volume-diffusion controlled nucleation the attachment frequency, f^*, is the product of the diffusion flux, j^*, of monomers to the surface of the nucleus and the area, A^*, of this surface. For a spherical nucleus, the surface area can be expressed in terms of its volume, which in turn is given by the number of molecules, n*, and the molecular volume, υ_o, and thus gives A^* as: $4\pi\left(\frac{3\upsilon_0 n^*}{4\pi}\right)^{\frac{2}{3}}$. The diffusion flux j^* is DC/r, where r is the radius of the nuclei, giving j^* as: $DC\left(\frac{4\pi}{3\upsilon_0 n^*}\right)^{\frac{1}{3}}$, where D is the diffusivity ($m^2\ s^{-1}$) and C is the molecular concentration which here represents the driving force for diffusional mass transfer.[4] C = C_e *S where C_e is solubility (mol m^{-3}).[4] The attachment frequency, f^*, for volume-diffusion controlled nucleation, then becomes:

$$f^* = (48\pi^2 \upsilon_o)^{\frac{1}{3}} D C_e S n^{*\frac{1}{3}} \quad \textbf{[5]}$$

For the formation of a cocrystal nucleus the diffusion can be limited by the transport of one of the components and in that case D, C and Ce are for that particular component.

For interface-transfer controlled nucleation f^* is again the product of the flux, j^*, of monomers and A^*. However in this case the flux, j^*, describes the mass transfer into the surface site by molecular jumps from a nearby site and is given as DC/d_{MT}, where d_{MT} is the distance of the molecular jump assumed to be approximated by the molecular diameter of the molecule that is governing the mass transfer process. d_{MT} is expressed in terms of the corresponding molecular volume, υ_{MT}, as $(6\upsilon_{MT}/\pi)^{1/3}$. The attachment frequency for the interface-transfer controlled nucleation then becomes:

$$f^* = \left(6\pi^2 \frac{{\upsilon_o}^2}{\upsilon_{MT}}\right)^{\frac{1}{3}} DC_e S n^{*\frac{2}{3}} \qquad [6]$$

The Zeldovich factor is given by **Eq [7]**[4]:

$$\mathrm{z} = \left(\frac{W^*}{3\pi kTn^{*2}}\right)^{\frac{1}{2}} \qquad [7]$$

and only contains molecular volumes that relates to the thermodynamics of the nucleation as per **Eqs [8]** and **[9]**. n^* is number of molecules in the critical nucleus and W^* is the nucleation work (J), as given below

$$n^* = \frac{32\pi\upsilon_o^2\gamma^3}{3(kTlnS)^3} \qquad [8]$$

$$W^* = \frac{1}{2}n^*kTlnS \qquad [9]$$

Eq [8] is based on a thermodynamic analysis of the free energy of the critical nucleus, and accordingly the number of molecules, n^*, is associated with a corresponding molecular volume. The molecular volume, υ_o (m^3) in **Eq [8]** is the volume occupied by a cluster/molecule in the nucleus and is associated with how a molecule is defined in the driving force expression, $kT\ln S$. It is the volume used to convert free energy per unit of cluster volume to free energy per molecule in the cluster. Depending on the definition used for a "molecule of cocrystal" the molecular volume will differ.

Inserting the above expressions given for z, f^* and C_o and substituting them into **Eq [3]** gives the expressions for the preexponential factor, A, for nucleation controlled by volume-diffusion, **Eq [10]**, and interface-transfer, **Eq [11]**. In the volume-diffusion case, **Eq [10]**, the molecular volume in f^* originates from the nucleus size and thus has the thermodynamic meaning, which leads to that this parameter cancels out and only the molecular volume of the solvent V_o term from **Eq [4]** remains**.** In case of attachment controlled by interface-transfer all three different molecular volumes remain in the equation as given by **Eq [11]**.

$$A = \left(\frac{kT}{\gamma}\right)^{\frac{1}{2}} \frac{1}{V_o} D C_e lnS \qquad \textbf{[10]}$$

$$A = \left(\frac{4\pi}{3\upsilon_{MT}}\right)^{\frac{1}{3}} \left(\frac{\gamma}{kT}\right)^{\frac{1}{2}} \frac{1}{V_0} \upsilon_0 D_s C_e \qquad \textbf{[11]}$$

Is nucleation of the cocrystal more difficult?

The units of *S* for the pure compounds are mole fraction/mole fraction and the units for the cocrystal are in terms of (mole fraction/mole fraction) squared although the units obviously cancel out in both cases. In order to have equal dimensionality, the square root of *S* from **Eq [12]:**

$$S = \frac{X^A . X^B}{(X^A . X^B)^*} \qquad \textbf{[12]}$$

giving *S* as **Eq [13]** can be used which changes the numerical value, **Table 8**.

$$S = \left(\frac{\sqrt{X_a X_b}}{\sqrt{(X_a X_b) *}}\right) \qquad \textbf{[13]}$$

Table 8 Different *S* values for cocrystal systems.

PHBA:GLU			THP:SA	
**Soluiton**	*S*, original[a]	*S*, new[b]	*S*, original[a]	*S*, new[b]
**1:1**	1.45	1.20	1.46	1.21
	1.59	1.26	1.57	1.25
	1.61	1.27	1.77	1.33
	1.64	1.28	2.16	1.47
	1.66	1.29	2.25	1.50
	1.72	1.31	2.56	1.60
**E1**	1.51	1.23	2.89	1.70
	1.64	1.28		
	1.72	1.31		
	1.94	1.39		
	2.27	1.51		
**E2**	1.36	1.17		
	1.40	1.18		
	1.45	1.20		
	1.69	1.30		
	1.86	1.36		
	1.93	1.39		

[a] *S* as per Eq [2] main manuscript

[b] *S* as $\left(\frac{\sqrt{X_a X_b}}{\sqrt{(X_a X_b)*}}\right)$, **Eq [13]**

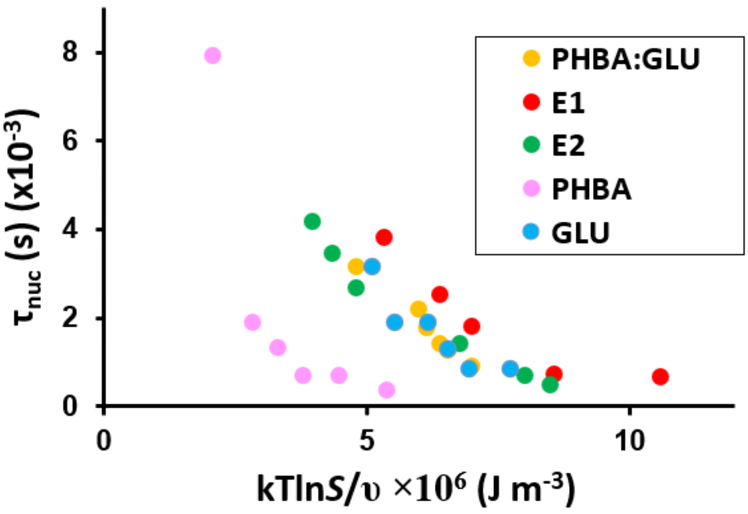

Figure 29 Nucleation difficulty in terms of free energy per unit volume to reach τ_{nuc} is the same outcome as defining driving force per reactant molecule.

GLU dimers in binary system

From the pure solutions, PHBA nucleates easier than β-GLU, Figure 10 main manuscript, and this is captured by a higher experimentally determined pre-exponential factor and a lower interfacial energy. When *A* is calculated according to the theoretical expressions however, the *A* value for GLU is higher than for PHBA irrespective of mechanism, mainly related to that the equilibrium concentration is higher for β-GLU. However, GLU has been reported to form cyclic dimers in solution with aprotic solvents, **Figure 30**.[6,7] Assuming GLU-dimers to be the unit governing the transport the molecular volume would be approximately twice that of GLU monomers giving the υ_o term in Eqs [6] and [7] main manuscript a value of 3.10×10^{-28} m^3, and according to Eq [8] main manuscript *D* decreases from 1.015 to 0.670×10^{-10} m^2 s^{-1}. The resulting *A* values are given in

Table 9 9. In the calculations the equilibrium concentration is taken as that of the monomer since the lower dimer concentration is fully balanced by the fact that each unit is carrying two molecules.

Table 9 Pre-exponential factor, *A*, calculated for nucleation in the pure GLU and pure PHBA systems according to Eq Error! Reference source not found.8] volume-diffusion c ontrolled nucleation and Eq [9] interface-transfer controlled nucleation. C_e is in mol m^{-3} and the *A* values are x10^{10} m^{-3} s^{-1}.

**β-GLU (dimers)**[a]			
**S**	Ce	A, Volume-diffusion	A, Interface-transfer
**1.22**	586	3.24	7.24
**1.24**	586	3.51	7.24
**1.27**	586	3.90	7.24
**1.29**	586	4.15	7.24
**1.31**	586	4.41	7.24
**1.35**	586	4.90	7.24

[a] Calculated assuming GLU forms dimers in solution.

The *A* values calculated by assuming GLU forms dimers in solution gives values similar to those obtained for the pure PHBA system, while PHBA experimentally has a higher pre-exponential factor than GLU - 153 m^{-3} s^{-1}versus 126 m^{-3} s^{-1}. Notably, the theoretical expressions are based on that transport rate is the governing factor while in reality conformational changes and specific desolvation effects can be important. For example, in order to integrate into the β-GLU crystal lattice the GLU cyclic dimer intermolecular bonds have to be broken, adding additional resistance to lattice integration.

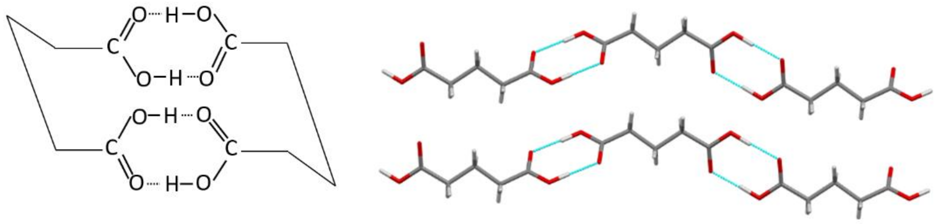

Figure 30 Left: Cyclic dimerization of GLU in solution.[7] Right: β-GLU crystal lattice.

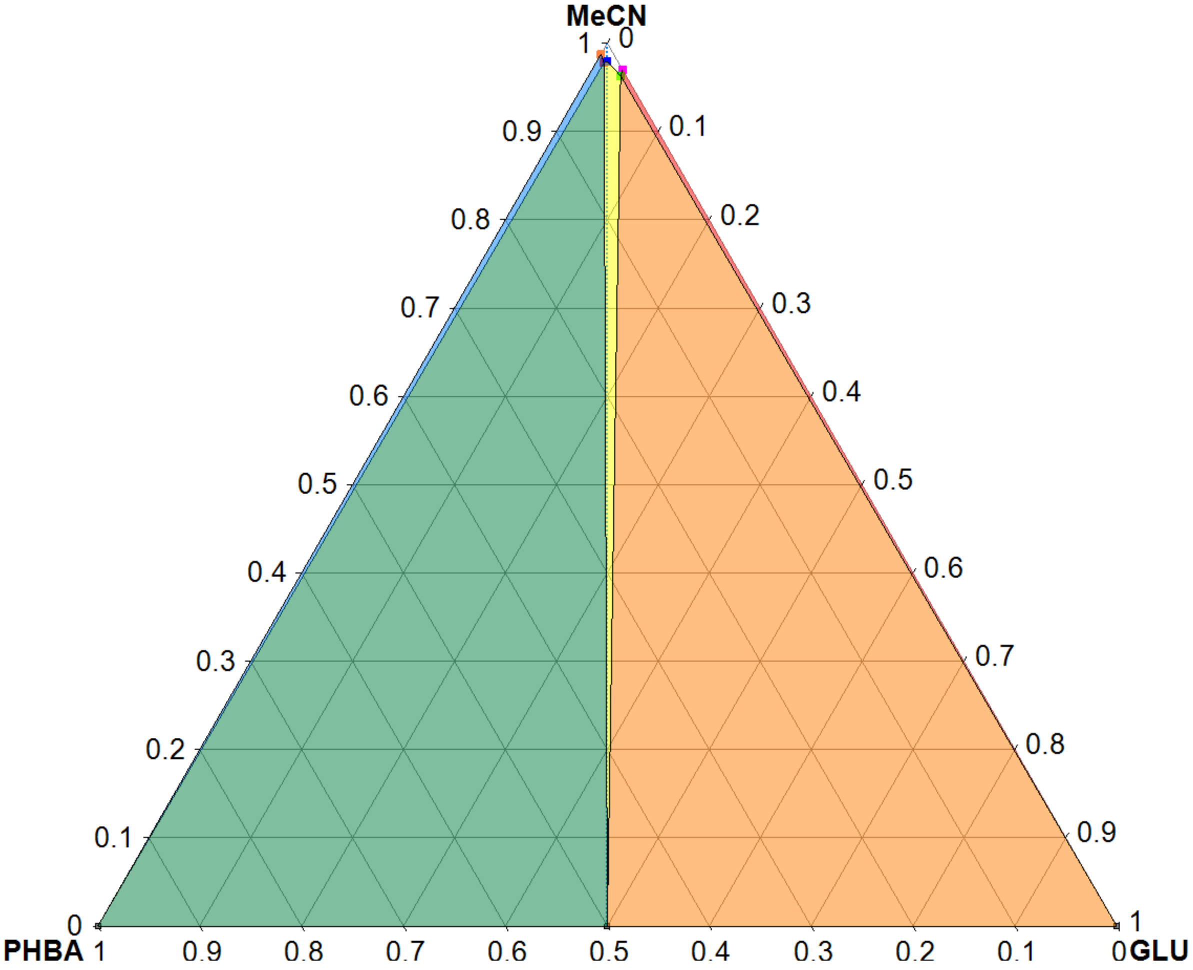

Figure 31 Ternary Phase Diagram for PHBA:GLU 1:1 cocrystal at 20 °C in MeCN, compositions in mole fraction.

Bibliography

(1) Yang, J.; Hong, B.; Wang, N.; Li, X.; Huang, X.; Bao, Y.; Xie, C.; Hao, H. Thermodynamics and Molecular Mechanism of the Formation of the Cocrystals of: P-Hydroxybenzoic Acid and Glutaric Acid. *CrystEngComm* **2019**, *21* (42), 6374–6381. https://doi.org/10.1039/c9ce01092k.

(2) Heath, E. A.; Singh, P.; Ebisuzaki, Y. Structure of P-Hydroxybenzoic Acid and p-Hydroxybenzoic Acid-Acetone Complex (2/1). *Acta Crystallogr. Sect. C Cryst. Struct. Commun.* **1992**, *48* (11), 1960–1965. https://doi.org/10.1107/s0108270192002361.

(3) McTague, H.; Rasmuson, Å. C. Nucleation of the Theophylline:Salicylic Acid 1:1 Cocrystal. *Cryst. Growth Des.* **2021**, *21* (5), 2711–2719. https://doi.org/10.1021/acs.cgd.0c01594.

(4) Kashchiev, D.; Van Rosmalen, G. M. Review: Nucleation in Solutions Revisited. *Cryst. Res. Technol.* **2003**, *38* (7–8), 555–574. https://doi.org/10.1002/crat.200310070.

(5) Nielsen, A. E. Nucleation and Growth of Crystals at High Supersaturation. *Krist. und Tech.* **1969**, *4* (1), 17–38. https://doi.org/10.1002/crat.19690040105.

(6) Gerken, J. B.; Badger, C.; Bisbee, C.; Gardner, S.; Qi, Y.; Vilà, V. D.; Roberts, J. D. Solution Conformational Preferences of Glutaric, 3-Hydroxyglutaric, 3-Methylglutaric Acid, and Their Mono- And Dianions. *J. Phys. Org. Chem.* **2008**, *21* (3), 193–197. https://doi.org/10.1002/poc.1277.

(7) Takasuka, M.; Saito, T.; Yamakawa, M. FTIR Spectral Study of Double Cyclic Intermolecular Hydrogen Bonding in Glutaric Acids. *J. Chem. Soc. Perkin Trans. 2* **1991**, No. 10, 1513–1516. https://doi.org/10.1039/p29910001513.
